# Deconvolution of expression for nascent RNA-sequencing data (DENR) highlights pre-RNA isoform diversity in human cells

**DOI:** 10.1093/bioinformatics/btab582

**Published:** 2021-08-11

**Authors:** Yixin Zhao, Noah Dukler, Gilad Barshad, Shushan Toneyan, Charles G Danko, Adam Siepel

**Affiliations:** Simons Center for Quantitative Biology, Cold Spring Harbor Laboratory, Cold Spring Harbor, NY 11724, USA; Simons Center for Quantitative Biology, Cold Spring Harbor Laboratory, Cold Spring Harbor, NY 11724, USA; Baker Institute for Animal Health, College of Veterinary Medicine, Cornell University, Ithaca, NY 14853, USA; Department of Biomedical Sciences, College of Veterinary Medicine, Cornell University, Ithaca, NY 14853, USA; Simons Center for Quantitative Biology, Cold Spring Harbor Laboratory, Cold Spring Harbor, NY 11724, USA; Baker Institute for Animal Health, College of Veterinary Medicine, Cornell University, Ithaca, NY 14853, USA; Department of Biomedical Sciences, College of Veterinary Medicine, Cornell University, Ithaca, NY 14853, USA; Simons Center for Quantitative Biology, Cold Spring Harbor Laboratory, Cold Spring Harbor, NY 11724, USA

## Abstract

**Motivation:**

Quantification of isoform abundance has been extensively studied at the mature RNA level using RNA-seq but not at the level of precursor RNAs using nascent RNA sequencing.

**Results:**

We address this problem with a new computational method called Deconvolution of Expression for Nascent RNA-sequencing data (DENR), which models nascent RNA-sequencing read-counts as a mixture of user-provided isoforms. The baseline algorithm is enhanced by machine-learning predictions of active transcription start sites and an adjustment for the typical ‘shape profile’ of read-counts along a transcription unit. We show that DENR outperforms simple read-count-based methods for estimating gene and isoform abundances, and that transcription of multiple pre-RNA isoforms per gene is widespread, with frequent differences between cell types. In addition, we provide evidence that a majority of human isoform diversity derives from primary transcription rather than from post-transcriptional processes.

**Availability and implementation:**

DENR and nascentRNASim are freely available at https://github.com/CshlSiepelLab/DENR (version v1.0.0) and https://github.com/CshlSiepelLab/nascentRNASim (version v0.3.0).

**Supplementary information:**

Supplementary data are available at *Bioinformatics* online.

## 1 Introduction

For about the last 15 years, most large-scale transcriptomic studies have relied on high-throughput short-read sequencing technologies as the readout for the relative abundances of RNA transcripts (Wang *et al.*, 2009). In species with available genome assemblies, these sequence reads are generally mapped to assembled contigs, and then the ‘read depth’, or average density of aligned reads, is used as a proxy for the abundance of RNAs corresponding to each annotated transcription unit. The approach is relatively inexpensive and straightforward, and, with adequate sequencing depth, it generally leads to accurate estimates of abundance (Conesa *et al.*, 2016; Corchete *et al.*, 2020).

A fundamental challenge with this general paradigm, however, is that transcription units frequently overlap in genomic coordinates—i.e. the same segment of DNA often serves as a template for multiple distinct RNA transcripts. As a result, it is unclear which transcription unit is the source of each sequence read. While this problem can occur at the level of whole genes that contain overlapping segments, it is most prevalent at the level of multiple isoforms for each gene, owing to alternative transcription start sites (TSSs), alternative polyadenylation and cleavage sites (PASs) and alternative splicing (Wang *et al.*, 2008). These isoforms often overlap heavily with one another, and differ on a scale that is not well described by short-read sequencing. This problem is critical because the existence of multiple isoforms per gene is the rule rather than the exception in most eukaryotes. For example, more than 90% of multi-exon human genes undergo alternative splicing (Wang *et al.*, 2008), with an average of more than seven isoforms per protein-coding gene (Zhang *et al.*, 2017); in plants, up to 70% of multi-exon genes show evidence of alternative splicing (Chaudhary *et al.*, 2019).

In the case of RNA-seq data, the problem of isoform-abundance estimation from short-read sequence data has been widely studied for more than a decade (Jiang and Wong, 2009; Katz *et al.*, 2010; Trapnell *et al.*, 2010). Several software packages now address the problem efficiently and effectively, including ones that make use of fully mapped reads (Li and Dewey, 2011; Roberts and Pachter, 2013) and others that substantially boost speed by working only with ‘pseudoalignments’ at remarkably little (if any) cost in accuracy (Bray *et al.*, 2016; Patro *et al.*, 2014, 2017). These computational methods differ in detail but they generally work by modeling the observed sequence reads as an unknown mixture of isoforms at each locus. They estimate the relative abundances (mixture coefficients) of the isoforms from the read-counts, relying in particular on the subset of reads that reflect distinguishing features, such as exons or splice junctions present in some isoforms but not others. Because RNA-seq libraries are typically dominated by mature RNAs, intronic reads tend to be rare and splice junctions provide one of the strongest signals for differentiation of isoforms. Altogether, these isoform quantification methods work quite well, with the best methods exhibiting Pearson correlation coefficients of 0.95 or higher with true values in simulation experiments, and similarly high concordance across technical replicates for real data (Zhang *et al.*, 2017).

In recent years, another method for interrogating the transcriptome, known as ‘nascent RNA sequencing’, has become increasingly widely used. Instead of measuring the concentrations of mature RNAs, as RNA-seq effectively does, nascent RNA-sequencing protocols isolate and sequence newly transcribed RNA segments, typically by tagging them with selectable ribonucleotide analogs or through isolation of polymerase-associated RNA (Churchman and Weissman, 2011; Core *et al.*, 2008; Duffy *et al.*, 2018; Kwak *et al.*, 2013; Mayer *et al.*, 2015; Michel *et al.*, 2017; Schwalb *et al.*, 2016). In this way, they provide a measurement of primary transcription, independent of the RNA decay processes that influence cellular concentrations of mature RNAs. In addition, nascent RNA-sequencing methods have a wide variety of other applications, including identification of active enhancers (through the presence of eRNAs) (Core *et al.*, 2014; Danko *et al.*, 2015; Michel *et al.*, 2017), characterization of promoter-proximal pausing and divergent transcription (Churchman and Weissman, 2011; Core *et al.*, 2008), estimation of elongation-rates (Danko *et al.*, 2013; Jonkers *et al.*, 2014) and estimation of relative RNA half-lives (Blumberg *et al.*, 2021). In this article, we focus in particular on the Precision Run-On sequencing (PRO-seq) protocol, which allows engaged polymerases to be mapped genome-wide at single-nucleotide resolution.

In nascent RNA sequencing, the isolated RNAs have generally not yet been spliced; therefore, they represent the entire transcribed portion of the genome, including introns. As a result, the problem of distinguishing alternative splice forms is largely irrelevant. On the other hand, the data typically still reflect a mixture of precursor RNA (pre-RNA) isoforms, having different TSSs and/or PASs. Moreover, the problem of decomposing this mixture can be more challenging than for RNA-seq in some respects, both because pre-RNA isoforms have fewer differentiating features than mature RNA isoforms, and because nascent RNA read depths tend to be substantially reduced, since introns as well as exons are sequenced. Distinguishing among pre-RNA isoforms in nascent RNA sequence data can be critical for a wide variety of downstream analyses (Blumberg *et al.*, 2021; Dukler *et al.*, 2017; Siepel, 2021). Nevertheless, to our knowledge, only one computational tool has been developed to address this problem—a program called TuSelector that was introduced by Dukler *et al.* (2017)—and it has never been packaged for use by other research groups or rigorously evaluated for accuracy. In most analyses of nascent RNA-sequencing data, the isoform deconvolution problem is either ignored or addressed by simple heuristics, such as assuming each gene is represented by the longest annotated isoform (Vaid *et al.*, 2020; Xiao *et al.*, 2019).

In this article, we introduce a new computational method and implementation in R, called Deconvolution of Expression for Nascent RNA-sequencing data (DENR), that addresses the problem of isoform-abundance quantification at the pre-RNA level. DENR also solves the closely related problems of estimating abundance at the gene level, summing over all isoforms, and identifying the ‘dominant isoform’, i.e. the one exhibiting the greatest abundance. DENR makes use of a straightforward non-negative least-squares strategy for decomposing the mixture of isoforms present in the data, but then improves on this baseline approach by taking advantage of machine-learning predictions of TSSs and an adjustment for the typical shape-profile in the read-counts along a transcription unit. We show that the method performs well on simulated data, and then use it to reveal a high level of diversity in the pre-RNA isoforms inferred from PRO-seq data for several human cell types, including K562, CD4^+^ T-cells and CD14^+^ monocytes.

## 2 Materials and methods

### 2.1 Estimating isoform abundance

DENR estimates the abundance of each isoform by non-negative least-squares optimization, separately at each cluster. For a given cluster of *n* isoforms spanning *m* genomic bins, let $\beta =(\beta_{1},\ldots ,\beta_{n})\prime$ be a column vector representing the coefficients (weights) assigned to the isoforms, let $Y=(y_{1},\ldots ,y_{m})\prime$ be a column vector representing the read-counts in the bins, and let X be an* m* × n design matrix such that $x_{i,j}=1$ if isoform *j* spans bin i and $x_{i,j}=0$ otherwise (Supplementary Fig. S1). DENR estimates β such that,

(1) $$\hat{\beta }=\underset{\beta }{\arg\;\min}\;\underset{}{{\left(Y-X\mathrm{X\beta}\right)}^{T}(Y-X\mathrm{X\beta}),}\;\;\;$$

subject to the constraint that $\beta_{i}\geq 0$ for all i∈{1,…,n}. If the option to apply a log-transformation is selected, then the transformation is applied to both the elements of Y and those of Xβ, and the optimization otherwise proceeds in the same manner. In either case, DENR optimizes the objective function numerically using the BFGS algorithm with a boundary of zero for the *β_i_* values. Notice that, when the shape-profile correction is applied, the non-zero values in the design matrix X are adjusted upward and downward from 1 (see below).

After obtaining estimates for all isoform abundances *β_i_*, we normalize them by the total library depth to facilitate comparisons between samples. Isoform-level abundances are then converted to gene-level abundances by summing over all isoforms associated with each gene.

### 2.2 Machine-learning predictor for active TSSs

To distinguish active and inactive TSSs based on patterns of bidirectional transcription in nascent RNA-sequencing data, we implemented a convolutional neural network (CNN) classifier using the Keras interface to TensorFlow (Gulli and Pal, 2017). While other tools exist for this purpose (Azofeifa and Dowell, 2017; Danko *et al.*, 2015), we sought to integrate a lightweight predictor directly into DENR. We trained the CNN on previously published PRO-seq data from K562 cells (Dukler *et al.*, 2017), using matched GRO-cap data to identify positive and negative examples (Core *et al.*, 2014). GRO-cap is an adaptation of Global Run-On sequencing that enriches for 5′-7meGTP-capped RNAs and identifies active TSSs with high sensitivity and precision. We conservatively defined candidate TSSs as ‘active’ if they overlapped GRO-cap peaks from the HMM-based predictor described in Core *et al.* (2014), selecting the TSS with the maximum GRO-cap signal per peak. We defined candidates as ‘inactive’ if they did not overlap any such peaks, and did not fall near other active TSSs (≤100 bp) or mapped GRO-cap reads (≤25 bp) (Supplementary Fig. S2). The CNN was composed of a single 1-D convolutional layer, followed by a ReLU activation function, max-pooling and drop-out. The output was then flattened and fed into a densely connected layer, and finally a single sigmoid function was used to classify the TSS (Supplementary Fig. S3). The model was applied to feature vectors corresponding to strand-specific read-counts in 21 bins of width 51 bp, centered on the positive and negative strands; the 42 raw read-counts for each example were transformed to *z*-scores for scale-independence. The CNN was trained using the Adam optimizer (Kingma and Ba, 2017) with early stopping.

When the optional TSS-calling feature is in use, only isoforms corresponding to predict active TSSs are allowed to have non-zero weights. However, because the TSS predictor inevitably misses some active TSSs, DENR makes use of a heuristic method to identify and reconsider regions of ‘unexplained’ high-density polymerase. Specifically, an upstream polymerase ratio (UPR) statistic is calculated by taking the ratio of the read-count density inside the isoform (+0.5 to +2 kb relative to the TSS) to the density upstream of the isoform (–3 to –0.5 kb relative to the TSS; Supplementary Fig. S4). If the UPR of an isoform is ≥5, and there are no other active isoforms within 5 kb upstream or 6 kb downstream of its TSS, then the isoform is eligible to be assigned a non-zero weight.

### 2.3 Shape-profile correction

The shape-profile correction is empirically derived from a reference set of isoforms. Briefly, starting with the full set of annotations provided by the user, DENR identifies a subset of isoforms that, according to various heuristics, appear to be sufficiently long, robustly expressed and the sole source of sequencing reads in their genomic regions. DENR then tiles each representative isoform with bins of the user-specified size (default 250 bp), and maps those bins to a canonical [0, 1] interval. This mapping is intended to fix the scales of the promoter-proximal and termination regions, and allow the remaining gene-body to be compressed or expanded as needed. Specifically, the first 3 kb of each isoform is mapped (proportionally) to the interval [0, 0.2], the last 3 kb is mapped to [0.8, 1] and the remaining portion is mapped to the (0.2, 0.8) interval. Finally, the canonical shape-profile is obtained by averaging the relative read-count densities of the entire [0,1]-rescaled reference set of isoforms, using a loess fit for smoothing, and scaling the density such that the median value across the entire interval is one. This shape-profile is then used to adjust the design matrix X (see above) by replacing each value of one with the relative density at the corresponding location in the canonical shape-profile. The isoform weights are then estimated by least-squares, as usual. In the case of isoforms of length l≤6 kb, the first 0.75 *l* and last 0.25 *l* base-pairs are proportionally mapped to the [0, 0.2] and [0.8, 1.0] intervals, respectively, in the canonical shape-profile, and the interval (0.2, 0.8) is ignored. As an example, the shape-profile for a set of isoforms based on PRO-seq data from K562 cells (Dukler *et al.*, 2017) is shown in Supplementary Figure S5. Note that, while we sometimes refer to a ‘U-shape correction’, the ‘U’ shape is not assumed but is derived from the data.

### 2.4 Simulation of nascent RNA-sequencing data

Our non-parametric simulator for nascent RNA-sequencing data, called nascentRNASim, makes use of a template set of isoform annotations and a designated collection of well-defined isoform ‘archetypes’ and corresponding read-counts. The archetypes are selected as cases where the observed read-counts can be attributed to a single isoform (see below). Given these inputs, we simulate a synthetic dataset in five steps. First, we group the isoform annotations into non-overlapping strand-specific clusters, as in a DENR analysis. Second, we sample randomly (with resampling) from this set of clusters, and similarly, from the set of inter-cluster distances. Third, within each sampled cluster, we substitute for each isoform the archetype that is closest to it in genomic length, keeping the TSS at its original position relative to the beginning of the cluster. Fourth, we sample a new overall isoform abundance for each synthetic isoform from a distribution fitted by kernel density estimation to isoform-abundance estimates from GTEx for skeletal muscle (Lonsdale *et al.*, 2013). Finally, we obtain a new read-count for each position along the isoform by resampling from the original value in proportion to the simulated abundance estimate. In this way, we sample a full synthetic dataset, consisting of realistic clusters, each with a realistic distribution of isoforms and realistic patterns of read-counts, but with a known abundance for each isoform.

In this work, we used the PRO-seq dataset from Dukler *et al.* (2017) as our source dataset, together with isoforms from Ensembl (Supplementary Fig. S6). We selected a set of 62 archetypes manually, looking for isoforms with a range of lengths that exhibited relatively high read depth, appeared to be solely responsible for the local PRO-seq signal (i.e. they did not overlap other active isoforms and were at least ∼5 kb from other active genes), and showed a PRO-seq signal that approximately coincided with the annotated TSS and PAS, dropping to background levels nearby. We also considered GRO-cap data from Core *et al.* (2014) in identifying TSSs. Notice that the design of the simulator ensures that every synthetic isoform has the same length and approximate read-count pattern as one of the 62 archetypes, but isoforms may overlap (with additive contributions to read-counts) in the synthetic data. In this way, we are able to produce quite rich and complex patterns of simulated data despite the use of a relatively small set of archetypes. To ensure that the number of archetypes was not a limiting feature in our analysis, we repeated our benchmarking experiments with a larger set of 145 archetypes and found that our results were largely unchanged.

### 2.5 Applying DENR to synthetic data

To benchmark DENR’s performance, nascentRNASim was first used to simulate PRO-seq read-counts for 1500 genes. To thoroughly examine the effects of optional features on performance, all combinations of optional features, i.e. with and without TSS prediction, shape-profile correction, log-transformation of read-counts and with various numbers (0, 1 or 4) of masked bins at both the 5′ and 3′ end of each isoform, were tested on the synthetic data, resulting in a total of 72 test schemes ($2^{3}\times 3^{2}$) (Supplementary Figs S7 and S8). The scheme with TSS prediction, shape-profile correction, log-transformation of read-counts, masking of one bin around the TSS and four bins around the PAS performed well at both the gene and isoform levels. Therefore, this combination was used for all subsequent analyses in synthetic and real data except where otherwise noted. The gene-level comparison was performed on the whole set of genes, and on two complementary subsets: one for which active isoforms predominately used an internal TSS, and one for which they used the 5′-most TSS for transcription. Genes were defined as using internal TSSs if their dominant isoforms were transcribed from a TSS at least 1 kb downstream from the 5′-most TSS annotation; otherwise they were defined as using the 5′-most TSS (Supplementary Fig. S9). At the isoform level, we compared the performance of DENR and the read-count-based (RCB) method for both dominant isoforms determined by true abundances in simulation, and longest isoforms determined by the annotations. To make the estimates comparable, we masked 250 bp downstream from TSS and 1000 bp upstream from the PAS when counting reads for the RCB method. To ensure that 1500 simulated genes were sufficient, we repeated our benchmarking experiments with 10 000 genes and found the results to be similar.

For the RCB method, the abundance of a gene or isoform *i* is estimated in transcripts per million as follows:

$$q_{i}^{\text{RCB}}=\frac{r_{i}\cdot 10^{6}}{f_{i}T},$$

where *r_i_* is number of reads mapped to the genomic region in question (corresponding either to an isoform or the union of isoforms associated with a gene), *f_i_* is the length of that region and, $T=\sum_{g\in G}\frac{r_{g}}{f_{g}},$ where *G* is the set of all genes in the simulation (Wagner *et al.*, 2012).

### 2.6 Applying DENR to real data

To prepare bigWig files as input for DENR, we first processed published K562 (Dukler *et al.*, 2017) and CD4^+^ T-cell (Danko *et al.*, 2018) PRO-seq libraries using the PROseq2.0 pipeline (https://github.com/Danko-Lab/proseq2.0) in single-end mode (Chu *et al.*, 2019). The human genome assembly (GRCh38.p13) and isoform annotations were downloaded from Ensembl (release 99) (Cunningham *et al.*, 2019). Annotations of protein-coding genes from the autosomes and X chromosome were used, excluding genes that overlapped on the same strand. To identify genes producing two or more pre-RNA isoforms with high confidence, only genes with robust expression (i.e. ranking at top 75% of all expressed genes) in K562 (*n* = 7732) and CD4^+^ T-cells (*n* = 7632) were retained for analysis. To survey predominant usage of internal TSSs for transcription, genes with dominant pre-RNA isoforms transcribed from internal TSSs 1 kb downstream from the 5′ most TSSs were identified and visualized using Gviz (Hahne and Ivanek, 2016).

To investigate the differences in dominant isoforms between K562 and CD4^+^ T-cells, mature RNA isoform annotations were first grouped together if the distances between their annotated TSSs were <1 kb. The longest isoform in each group was selected as the representative and used for estimating abundance. Inactive TSSs were predicted separately in K562 and CD4^+^ T-cells and then intersected, to ensure that the same set of inactive isoforms was used across cell types. To identify genes with different dominant isoform between cell types, 6757 genes exhibiting robust expression (i.e. ranking in the top 75% in both cell types) were analyzed. We focused on cases in which the dominant isoforms differed in the two cell types. The Gene Ontology (GO) analysis was performed using the online tool DAVID (Huang *et al.*, 2009).

### 2.7 Calculation and decomposition of Shannon entropy

We made use of Shannon entropy as a general measure of isoform diversity. Let *X_i_* be a random variable representing the possible pre-RNA isoforms of gene *i*, and assume the probability density function for *X_i_* is proportional to DENR-based estimates of isoform abundance. That is, $p(X_{i}=j)=\frac{1}{Z_{i}}q_{ij}$, where *q_ij_* is the estimated abundance of the *j*th isoform of gene *i* and $Z_{i}=\sum_{j}q_{ij}$. We calculate the Shannon entropy of *X_i_* as $H(X_{i})=-\sum_{j}p(X_{i}=j){\;\text{log}\;}_{2}p(X_{i}=j)$, and we calculate the total entropy of a set of genes *S* as $H(X_{S})=\sum_{i\in S}H(X_{i})$, assuming independence of genes.

Similarly, let *Y_i_* represent the possible mature RNA isoforms of gene *i*, with $p(Y_{i}=k)=\frac{1}{Z\prime_{i}}q\prime_{ik}$, where $q\prime_{ik}$ is the StringTie-estimated abundance of the *k*th isoform of gene *i* and $Z\prime_{i}=\sum_{k}q\prime_{ik}$. Then, $H(Y_{i})=-\sum_{k}p(Y_{i}=k){\;\text{log}\;}_{2}p(Y_{i}=k)$, and, for a set of genes *S*, $H(Y_{S})=\sum_{i\in S}H(Y_{i})$.

To decompose entropy into components from *H*(*X*) (primary transcription) and H(Y|X) (post-transcriptional processes), we consider the joint entropy of *X* and* Y*, *H*(*X*, *Y*), and make use of the chain rule, H(Y|X)=H(X,Y)−H(X), interpreting H(Y|X) as the additional entropy contributed to the distribution of pre-RNA isoforms by post-transcriptional processes. Furthermore, because in this case, each mature RNA isoform corresponds to a single pre-RNA isoform, *H*(*X*, *Y*) is the same as *H*(*Y*). Specifically, for each *i*,

$$\begin{array}{c} H(X_{i},Y_{i})=-\sum_{j}\sum_{k\approx j}p(X_{i}=j,Y_{i}=k)\;{\text{log}}_{2}p(X_{i}=j,Y_{i}=k)\\ =-\sum_{k}p(Y_{i}=k)\;{\text{log}}_{2}p(Y_{i}=k)=H(Y_{i}), \end{array}$$

where k≈j indicates that mature RNA isoform *k* is compatible (in TSS and PAS) with pre-RNA isoform *j*. Thus, we estimate the post-transcriptional contribution as $H(Y_{i}|X_{i})=H(Y_{i})-H(X_{i})$.

## 3 Results

### 3.1 Overview of DENR

DENR is implemented as a package in the R programming environment. It requires two main inputs: a set of isoform annotations and a set of corresponding strand-specific nascent RNA-sequencing read-counts. Mature RNA isoform annotations can be easily downloaded by making use of biomaRt (Durinck *et al.*, 2005) or extracted from files in commonly available formats, such as GTF or GFF. Read-counts can be easily obtained from a file in bigWig format. Detailed examples are provided in the github repository (see Availability and implementation section).

**Fig. 1.**
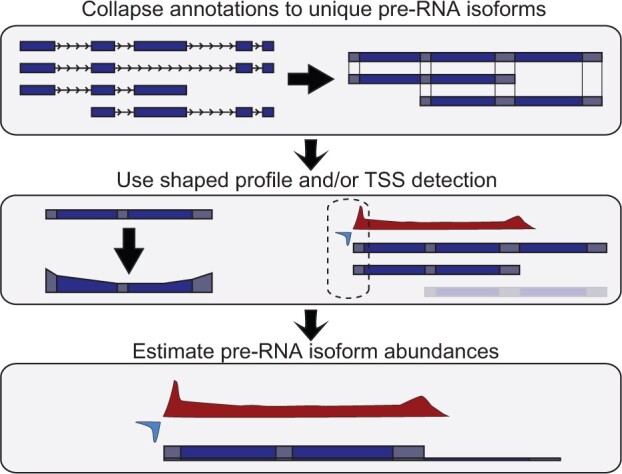
Illustration of DENR analysis. (*Top*) DENR first groups the available isoform annotations into non-overlapping, stand-specific clusters and summarizes the associated read-counts in genomic bins of user-specified size (default 250 bp). At this stage, it optionally masks bins corresponding to the start and end of each isoform. It then collapses mature RNA isoforms together that share start (TSS) and end (PAS) coordinates within the resolution of a single bin. (*Middle*) The program then optionally adjusts the isoform model to reflect a typical ‘U’-shaped profile, and optionally applies a machine-learning method to predict active TSSs based on patterns of bidirectional transcription. At this stage, it may also exclude isoforms designated by the user as inactive (not shown). (*Bottom*) Finally, DENR estimates the abundance of each isoform in each cluster by minimizing the squared difference between the expected and observed read-counts across all bins (see Section 2)

Given the necessary inputs, DENR first builds a *transcript_quantifier* object, which summarizes the read-counts corresponding to the available isoform annotations (Fig. 1 top panel). This phase consists of three steps (Supplementary Fig. S1). First, the mature RNA isoforms are grouped into non-overlapping, strand-specific clusters, corresponding roughly to genes (although if two genes overlap on the same strand, they will be grouped in the same cluster). Second, masking rules are applied to a user-specified number of bins, causing read-counts to be excluded at the start and end of each annotated isoform, to avoid the biases in quantification stemming from promoter-proximal pausing or termination-related deceleration of RNA polymerase. Third, the set of mature isoforms in each cluster is collapsed to a maximal set such that each isoform model has a unique pair of start and end coordinates, by merging all mature isoforms that share both their start and end bins. This step reduces isoforms annotated at the mature RNA level, many of which differ only in their splice patterns, to a more compact set of pre-RNA isoforms. It also merges pre-RNA isoforms that no longer differ from one another after masking. This second property is useful because the nascent RNA sequence data typically provides only approximate indications of the TSS and PAS associated with each transcript, owing to both sparseness of the data and imprecisions in the transcription process itself (such as transcriptional run-on at the 3′ end). The reduced set represents isoforms likely to be confidently distinguishable on the basis of nascent RNA sequence data alone. This set is recorded in the design matrix X for isoform-abundance estimation (Supplementary Fig. S1).

The second phase in a DENR analysis is, optionally, to provide auxiliary information that may improve the accuracy of isoform-abundance estimates. Any combination of three separate types of data can be provided: (i) the coordinates of predicted TSSs, (ii) a list of inactive isoforms and (iii) a shape-profile correction. Separate predictions of TSSs are useful because they help to distinguish the start of one isoform (particularly one downstream from the start of a cluster) from the continuation of another isoform. The DENR package includes a lightweight, pre-trained machine-learning classifier, implemented using TensorFlow, that can predict the locations of likely TSSs based on their characteristic patterns of bidirectional transcription and symmetric pause peaks (Section 2; Supplementary Figs S2 and S3). A separate specification of inactive isoforms can also be useful by directing the quantification algorithm to ignore a potentially large class of isoforms that may otherwise be misleading or confusing, based on auxiliary sources of data—including either experimental data, such as GRO-cap, PRO-cap, or RNA-seq, or computational predictions. The shape-profile correction is a way of accommodating the typical ‘U’-shaped profile of nascent RNA-sequencing reads along a gene-body, even after pause and termination peaks are excluded (Fig. 1 middle panel). This phenomenon is due to transcriptional pausing, acceleration of polymerase after pause escape and deceleration as the polymerase approaches the end of a gene (Kwak *et al.*, 2013; Wissink *et al.*, 2019). DENR also provides a function to estimate the average profile from a designated subset of the data, and then to consider its shape when estimating the abundance of each isoform (see Section 2).

Finally, DENR estimates the abundance of each isoform. Given the read-counts per bin for each isoform cluster, DENR simply estimates a weight for each isoform by least-squares, i.e. by minimizing the squared difference between the expected density and the observed read-count across all bins (see Section 2). An option is also provided to perform this optimization in logarithmic space, i.e. by comparing the logarithm of the expected density and the logarithm of the read-counts, corresponding to an assumption of a log-normal distribution for read-counts (see Section 4).

DENR is designed to be fast and efficient, and in our experiments on an Intel i7-10700 CPU (using a single thread) it was able to process a typical human dataset (∼20 000 genes, ∼25 million mapped reads) in about 10 min, with <8 GB of RAM. Notably, we used a bin size of 250 bp for all results reported in this article, finding that this size appropriately smoothed the raw PRO-seq signal and struck a good balance between genomic resolution and computational cost. However, we experimented with a smaller bin size of 125 bp and observed similar results (see below). For different datasets, users may wish to experiment with other bin sizes, ranging from, say, 50 to 500 bp.

### 3.2 DENR accurately estimates RNA abundance at the gene and isoform levels

We evaluated DENR’s accuracy in quantifying RNA abundance at both the gene and isoform levels. Lacking an appropriate ‘gold-standard’ in the form of real biological data, we chose to benchmark the software using simulated data. Because, to our knowledge, there is no available simulator for nascent RNA-sequencing data that accommodates multiple isoforms per gene, we developed a new R package, called nascentRNASim, to provide a ground truth against which to compare DENR’s estimates (Supplementary Fig. S6). To make the simulated data as realistic as possible, nascentRNASim makes use of an empirical distribution of relative isoform abundances per gene obtained from RNA-seq data from GTEx (Lonsdale *et al.*, 2013). Given this distribution, the program then generates synthetic nascent RNA-sequencing read-counts for each isoform by resampling PRO-seq read-counts from a manually curated set of archetypal isoforms (see Section 2). The read-counts from different isoforms are combined where they overlap. In this way, synthetic data are generated that closely resembles real data, without the need for restrictive modeling assumptions.

We first evaluated the impact of the various optional features by running the program with and without TSS prediction, shape-profile correction, log-transformation of read-counts and with various numbers (0, 1 or 4) of masked bins at the 5′ and 3′ ends of each isoform. We ran DENR on 1500 simulated loci, measuring the Pearson’s correlation coefficient (*r*) of the estimated and ‘true’ abundances at both the gene (Supplementary Fig. S7) and isoform (Supplementary Fig. S8) levels. We found, in general, that TSS prediction and the log transformation did indeed improve performance significantly at both the gene and isoform levels (all *P*-values <0.05, Wilcoxon test). The shape-profile correction also appeared to improve performance consistently at isoform level, although to a lesser extent (*P* = 0.149, Wilcoxon test). The effect of the masking strategy was more variable, but we found that masks of one bin at the 5′ end and four bins at the 3′ end performed best at the isoform level and were close to optimal at the gene level. Therefore, for simplicity, we used this masking strategy, and made use of TSS prediction, the shape-profile prediction, and the log-transformation at both the gene and isoform levels for all subsequent analyses on both simulated and real data.

**Fig. 2.**
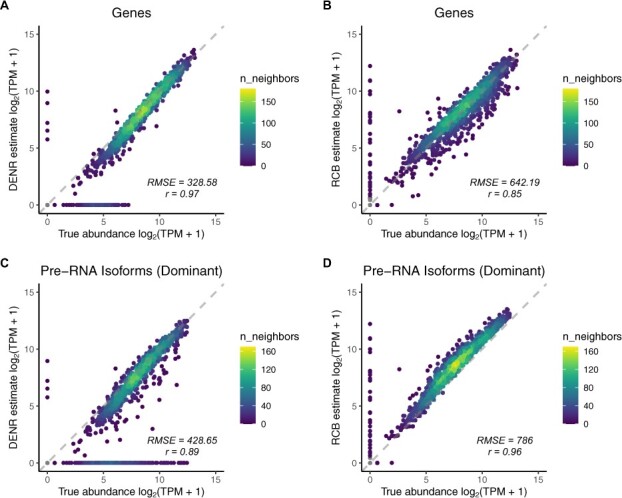
Comparison of DENR (left) and the simple RCB method (right) for quantifying nascent RNA abundance. True (*x*-axis) versus estimated (*y*-axis) abundance at the gene (**A and B**) and the ‘dominant’ isoform (most highly expressed; **C and D**) levels, based on 1500 simulated loci. Data were simulated using nascentRNASim, which resamples real PRO-seq read-counts and assumes a distribution of relative isoform abundances derived from real RNA-seq data. RMSE, root-mean-square error; *r*, Pearson’s correlation coefficient

With these options in place, we next compared DENR’s estimates for the same 1500 simulated loci with estimates obtained using a naive read-count-based (RCB) method commonly used in the field. For the RCB method, we simply estimated the abundance of a gene by the number of sequence reads that overlap any annotated isoform for that gene divided by the gene’s total length (see Section 2). At the gene level, DENR’s estimates were highly concordant with true abundances (*r* = 0.97) (Fig. 2A), substantially better than the RCB method (*r* = 0.85) (Fig. 2B). Accordingly, DENR exhibited much smaller root-mean-square error (RMSE = 328.6) than the RCB method (RMSE = 642.2) (Fig. 2A and B). DENR offered a particular improvement in cases where the dominant isoform corresponded to an internal TSS (Supplementary Fig. S9A), where the RCB method ‘over-normalized’ using the length of whole gene and therefore underestimated abundance (Supplementary Fig. S9B–D for comparison). However, several genes having non-zero true abundances were estimated to have values of zero by DENR (Fig. 2A), apparently owing to failures in TSS detection (see Section 4). The RCB method displayed the opposite tendency, estimating non-zero values for some genes having true values of zero (Fig. 2B). These cases were predominantly caused by overlap with or transcriptional run-on from other expressed genes.

We also compared estimates from DENR and the RCB method with the true RNA abundances at the level of individual isoforms. We focused our evaluation on a single isoform per gene, selecting either the most abundant—or ‘dominant’—isoform, as determined by the true abundances; or the longest isoform, as determined by the annotations (see Section 2). At the isoform level, DENR’s estimates of abundance were still well correlated with the true values (*r* = 0.89) (Fig. 2C), although, not surprisingly, the concordance was somewhat reduced compared with the gene-level analysis (Fig. 2A). The estimates from the RCB method showed high correlation with true abundances (*r* = 0.96) (Fig. 2D), but these estimates were systematically inflated, leading to substantially larger error (RMSE = 786.0) than that from DENR (RMSE = 428.7). This problem became more severe for the longest isoform, where DENR outperformed the RCB method substantially in terms of both correlation (*r* = 0.89 versus 0.59) and RMSE (297.5 versus 1117.3) (Supplementary Fig. S10). These biases occur because the RCB method tends to misattribute sequence reads arising from other isoforms to the isoform in question. While other counting strategies could be devised, there is ultimately no good way to estimate isoform-specific abundance without simultaneously considering all candidate isoforms and all sequence reads (see Section 4). Finally, as validation, we tested DENR’s performance with a smaller bin size (125 bp) (Supplementary Fig. S11) and on an expanded set of 10 000 simulated loci generated from a larger set of 145 archetypes (Supplementary Fig. S12), and found that the relative performance with the RCB method was largely unchanged.

### 3.3 Application to real data for K562 and CD4^+^ T-cells

Having demonstrated that DENR has good power to recover true gene and isoform abundances in simulated data, we next applied it to real data from K562 (Dukler *et al.*, 2017) and CD4^+^ T-cells (Danko *et al.*, 2018). We focused our analysis on 7732 and 7632 genes that displayed robust expression (ranking at the top 75% of all expressed genes) in K562 and CD4^+^ cells, respectively. In K562 cells, we found that nearly half of these genes (3624 of 7732, or 46.9%) displayed evidence of expression at two or more isoforms (see Section 2), indicating frequent use of alternative TSSs or PASs (248 with alternative TSSs, 2213 with alternative PASs and 1163 with both). We observed a similar pattern in CD4^+^ cells, with 48.9% (3734 of 7632) of genes producing two or more pre-RNA isoforms. Moreover, we found that the dominant isoforms for 1178 (15.2%) and 1262 (16.5%) of genes, respectively, made use of an internal TSS, at least 1 kb downstream from the 5′-most annotation.

To illustrate how DENR deconvolves the signal from PRO-seq data, we highlight two loci with multiple overlapping pre-RNA isoforms and evidence for internal TSS usage in K562 cells. The first example, at the gene *ST7*, is a relatively straightforward case (Supplementary Fig. S13). This gene has 30 (mature RNA) isoform annotations in Ensembl, which DENR merged into 19 distinct pre-RNA isoforms. However, the PRO-seq signal in the region suggests that only a subset of these isoforms are expressed, with clear signals beginning at a TSS near the 5′ end of the locus and at a second TSS about 60 kb downstream. Indeed, DENR estimated non-zero abundance for only two isoforms, with the shorter one (G14406M1, corresponding to five Ensembl isoforms; see Supplementary Table S1) obtaining a higher weight than the longer one (G14406M6, corresponding to two Ensembl isoforms); the remaining 17 isoforms were assigned weights of zero. Notice that the TSSs of both isoforms are clearly marked by bidirectional transcription in the PRO-seq data, a signal used by DENR in picking them out.

**Fig. 3.**
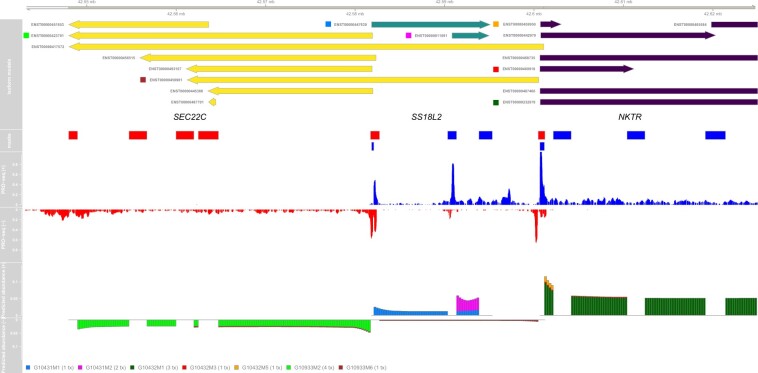
DENR abundance estimation for three overlapping genes on human chromosome 3. Isoform annotations are shown for *SEC22C* (yellow; ENSG00000093183), *SS18L2* (green; ENSG00000008324) and *NKTR* (purple; ENSG00000114857), together with the raw PRO-seq signal. The plot at *bottom* shows the expected relative contribution of each isoform model to the overall read-counts per bin. Notice the effect of the shape-profile adjustment near the 5′ and 3′ ends. Notice also that the PRO-seq data reveal bidirectional transcription near the TSSs of active isoforms; these signals are used by the machine-learning predictor to help identify sequence reads associated with these isoforms

The second example is a more complex case in which three expressed genes (*SEC22C*, *SS18L2* and *NKTR*) all overlap (Fig. 3). These genes all have multiple isoform annotations in Ensembl, some of which correspond to distinct pre-RNA isoforms after merging. In particular, *SEC22C* has 16 isoforms, which are merged into 8 pre-RNA isoforms; *SS18L2* has 3 isoforms, which are merged into 2; and *NKTR* has 19 isoforms, which are merged into 10. By again leveraging the signatures associated with TSSs, DENR identified two expressed isoforms of *SEC22C*, two expressed isoforms of *SS18L2* and three expressed isoforms of *NKTR*. In each case, one isoform is clearly dominant, although in the case of *SS18L2*, both are expressed at non-negligible levels (Supplementary Table S2). Notice that the dominant isoforms for both *SEC22C* and* SS18L2* make use of internal TSSs. Notice also that DENR attributes both expressed isoforms of *SEC22C* and the minor expressed isoform of *SS18L2* to the same TSS, suggesting that stable transcripts are generated bidirectionally from this site. A second TSS contributes bidirectionally to the dominant isoform of *NKTR* and a minor isoform of *SEC22C*.

### 3.4 Differences in dominant pre-RNA isoforms between CD4^+^ T-cells and K562 cells

**Fig. 4.**
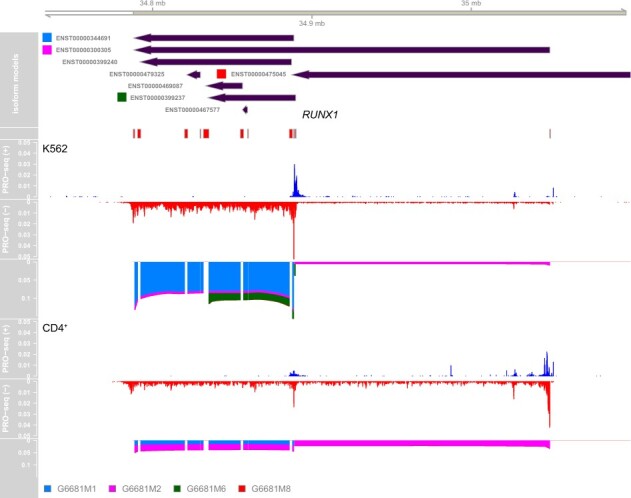
Cell-type-specific TSS usage for *RUNX1*. Of several annotated pre-RNA isoforms for the gene encoding the transcription factor *RUNX1* on human chromosome 21 (shown on the negative strand at *top*), DENR finds two isoforms to be dominant: a ∼100-kb isoform (G6681M1; shown in blue), and an isoform that is more than twice as long and begins ∼160 kb upstream (G6681M2; shown in pink). The shorter isoform is clearly dominant in K562 cells (*middle*), whereas both are expressed at non-negligible levels in CD4^+^ T-cells, with the longer one being slightly dominant (*bottom*). *RUNX1* is essential for normal hematopoietic development and its dysregulation is associated with hematological malignancies (Sood *et al.*, 2017). It is well known to make use of alternative promoters (de Bruijn and Dzierzak, 2017; Otálora-Otálora *et al.*, 2019)

Given DENR’s ability to identify dominant pre-RNA isoforms, we wondered how frequently these isoforms might differ between cell types. We therefore compared the predictions of dominant isoforms from K562 cells to those from CD4^+^ T-cells. Because the 3′ ends of pre-RNA transcription units can be difficult to pinpoint owing to transcriptional run-on, we focused on genes for which the dominant isoforms clearly used different TSSs in the two cell types, requiring a difference of at least 1 kb in genomic coordinates (see Section 2). In addition, we limited our analysis to 6757 genes showing robust expression (ranking in the top 75%) in both cell types. We found that 238 of these genes (∼3.5%) had dominant isoforms that made use of different TSSs in K562 and CD4^+^ T-cells. A GO analysis showed that these genes were significantly enriched for annotations of alternative splicing (Supplementary Fig. S14), suggesting a correlation between alternative TSS usage and alternative splicing. One prominent example in this group is the gene encoding the transcription factor *RUNX1*, a master regulator of hematopoietic stem cell differentiation (Fig. 4), which has a much longer dominant isoform—resulting from a TSS about 160 kb upstream—in CD4^+^ T-cells as compared with K562 cells. This gene is known to make use of alternative TSSs in a temporal and tissue-specific manner (de Bruijn and Dzierzak, 2017; Otálora-Otálora *et al.*, 2019; Sood *et al.*, 2017). Additional examples are shown in Supplementary Figure S15.

### 3.5 Relative contributions of transcriptional and post-transcriptional processes to isoform diversity

We were interested in making use of DENR to assess overall levels of isoform diversity genome-wide. Furthermore, we wondered if a parallel analysis of RNA-seq data would enable an informative comparison of the relative contributions to isoform diversity at the pre-RNA and mature RNA levels. Toward this end, we generated high-quality matched PRO-seq and RNA-seq datasets (both with paired-end reads; see Section 2) for two similar but distinct human cell types, CD4^+^ T-cells and CD14^+^ monocytes. We used DENR to quantify isoform abundance at the pre-RNA level and StringTie (Pertea *et al.*, 2016) to quantify isoform abundance at the mature RNA level in each cell type. To make the comparison as direct as possible, we directed DENR to ignore isoforms not detected at the RNA-seq level, instead of relying on the automatic TSS prediction feature. We focused our analysis on a set of 10 650 genes that were expressed in both cell types, with good representation in both the PRO-seq and RNA-seq datasets (see Section 2).

To quantify isoform diversity at the pre-RNA and mature RNA levels, we made use of the information-theoretic measure of Shannon entropy. We observed that, given pre-RNA isoform-abundance relative frequencies *X* (estimated from PRO-seq data using DENR) and mature RNA isoform-abundance relative frequencies *Y* (estimated from RNA-seq data using StringTie), the joint entropy *H*(*X*, *Y*) can be decomposed into a component arising from primary transcription, *H*(*X*), and a conditional-entropy component arising from post-transcriptional processes, H(Y|X); i.e. H(X,Y)=H(X)+H(Y|X) (see Section 2). Thus, we can estimate *H*(*X*) across any set of expressed genes using DENR, estimate *H*(*X*, *Y*) for the same set of genes using StringTie, and then estimate the post-transcriptional entropy, H(Y|X) by their difference. We can further estimate the fractional contribution of transcription to the final isoform entropy as H(X)/H(X,Y). In this way, we can quantify the relative contributions to isoform diversity of transcriptional and post-transcriptional processes.

**Fig. 5.**
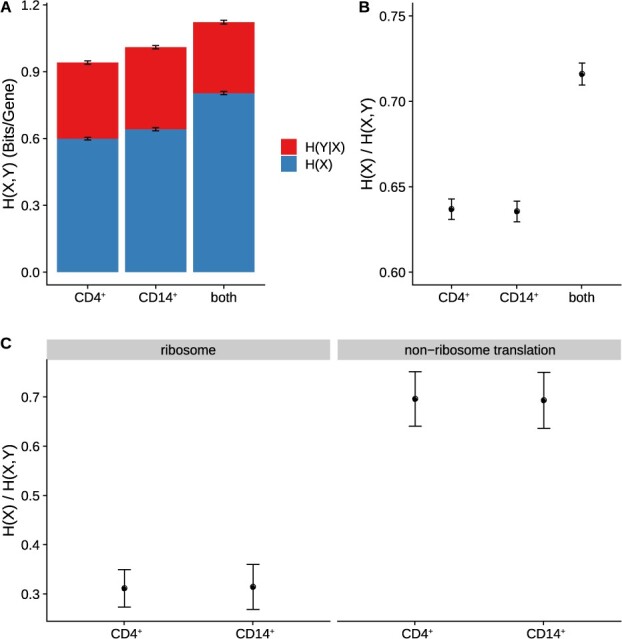
Decomposition of Shannon entropy of isoform diversity into contributions from primary transcription and post-transcriptional processing. (**A**) Entropy per gene of mature RNA isoforms [*H*(*X*, *Y*)] is partitioned into a component from primary transcription [*H*(*X*)] and a component from post-transcriptional processing, including splicing [H(Y|X)]. (**B**) Fractional contribution from primary transcription, H(X)/H(X,Y). Results are for 10 650 genes expressed in both CD4^+^ T-cells and CD14^+^ monocytes. ‘Both’ indicates results when both datasets are pooled. (**C**) Fractional contribution from primary transcription, as in (B), but for the subsets of genes associated with GO terms ‘ribosome’ (left, GO: 0005840; *n* = 135) and ‘translation’ (GO: 0006412) but not ‘ribosome’ (right; *n* = 119). Error bars represent the standard deviation of the mean as estimated by bootstrap resampling (*n* = 100)

When applying these methods to the CD4^+^ T-cell and CD14^+^ monocyte datasets individually, we observed reasonably good concordance, with estimates of *H*(*X*, *Y*) = 0.94–1.01 bits/gene in total entropy, of which a clear majority, 63–64%, comes from transcriptional entropy [*H*(*X*)] and the remaining 36–37% derives from post-transcriptional processes (Fig. 5A and B). When we pooled data from the two cell types together (‘both’), *H*(*X*, *Y*) increased by about 10%, indicating higher levels of isoform diversity across cell types than within them. Interestingly, however, the fractional contribution from primary transcription, H(X)/H(X,Y), also increased substantially, from ∼0.64 to ∼0.72, suggesting that transcriptional processes make a disproportional contribution to the isoform diversity across cell types, which is more likely than diversity within each cell type to be associated with true functional differences (see Section 4).

A primary difference between these cell types is that CD4^+^ T-cells play an important role in the adaptive immune system whereas CD14^+^ monocytes are part of the innate immune system. Therefore, we extracted 116 and 287 genes associated with the GO terms ‘adaptive immune response’ and ‘innate immune response’, respectively, and calculated H(X)/H(X,Y) separately for each of these subsets of genes. Interestingly, we found that this fraction was somewhat elevated in adaptive-immunity-related genes in CD4^+^ T-cells (Wilcoxon signed-rank test, *P* = 0.0002), and slightly elevated in innate-immunity-related genes in CD14^+^ monocytes (Supplementary Fig. S16; Wilcoxon signed-rank test, *P* = 8.59e-14), suggesting that primary transcription may disproportionally contribute to isoform diversity in the genes most relevant to the specific immune-related functions of each cell type. Examining several other classes of genes (Supplementary Fig. S17), we found that genes associated with the GO term ‘translation’ display a substantial reduction in H(X)/H(X,Y) compared with genes in the ‘transcription’, ‘RNA splicing’, and other GO categories. Further examination of ‘translation’ genes showed that the reduction was predominately driven by genes encoding ribosomes (Fig. 5C), with only ∼30% of isoform diversity coming from primary transcription, and the remaining ∼70% being contributed by post-transcriptional processes. These findings are consistent with previous reports that most ribosomal protein genes predominately used one or a few promoters across human tissues (Guimaraes and Zavolan, 2016), yet are strongly influenced by alternative splicing (Brumwell *et al.*, 2020; Song *et al.*, 2017).

## 4 Discussion

In this article, we have introduced DENR, the first fully vetted computational method—to our knowledge—to address the abundance estimation problem at the level of pre-RNA isoforms, based on nascent RNA-sequencing data. At its core, DENR is simply a regression-like method for estimating a weight for each element in a set of predefined candidate isoforms, by minimizing the sum-of-squares difference between expected and observed read-counts. This baseline model is augmented by various refinements, including machine-learning predictions of TSSs, a shape-profile correction for read-counts, and masking of read-counts near isoform TSSs and PASs. We have shown that DENR performs well on simulated and real data. We expect it to be useful in a variety of downstream applications, such as the identification of differentially expressed genes (Dukler *et al.*, 2017), RNA half-life estimation (Blumberg *et al.*, 2021), the study of transcription unit evolution (Danko *et al.*, 2018) and the identification of differential pause-release rates (Siepel, 2021).

In direct comparisons with simple RCB methods like those used in most current applications, we find that DENR does indeed offer a substantial performance improvement. The improvement is most pronounced at the isoform level, where the RCB methods inevitably misattribute many reads to the wrong isoform. Interestingly, however, DENR also improves substantially on gene-level estimates of abundance. The main reason for this improvement has to do with the normalization for gene length. The gene-level RCB method has no good way to identify which bases in the DNA template are transcribed, and must conservatively assume transcription occurs across the union of all annotated isoforms. As a result, it frequently ‘over-normalizes’ and underestimates abundance. DENR, by contrast, simultaneously models all isoforms and explains the full set of read-counts at a locus as a mixture of isoforms. The limitations we observed with alternative RCB methods highlight the difficulty of accurately estimating abundance without a model that assigns reads to isoforms in zero-sum fashion. Because most reads can potentially arise from multiple alternative isoforms, any naive counting method will tend to either over- or under-estimate abundance. These errors in abundance estimation, in turn, can result in biases in many downstream applications, such as elongation-rate or RNA-half-life estimation.

In analyses of real data, we found that many genes (nearly half of robustly expressed genes in K562 and CD4^+^ T-cells) display evidence of expression at multiple distinct pre-RNA isoforms. Moreover, we found that the dominant isoform fairly commonly (in ∼15% of cases) makes use of a TSS that is >1 kb downstream of the 5′-most annotation. These cases are particularly likely to be mischaracterized by standard methods for quantifying pre-RNA expression. We have highlighted specific examples showing how DENR can effectively deconvolve the read-count contributions of multiple overlapping isoforms, including a complex case involving multiple overlapping genes (Fig. 3). In addition, in a comparison of K562 and CD4^+^ T-cells, we identified more than 200 genes that use different dominant isoforms in these two cell types, including prominent examples, such as *RUNX1*.

One interesting consequence of having the ability—as we now do—to characterize the distribution of isoform abundances at both the pre- and mature RNA levels is that it potentially allows for a decomposition of the contributions to isoform diversity from primary transcription and post-transcriptional processes. In a final analysis, we attempted to quantify these relative contributions using a simple information-theoretic calculation, by partitioning the Shannon entropy in mature RNA isoform diversity (as estimated from RNA-seq data using StringTie) into a component estimated at the pre-RNA level (by applying DENR to PRO-seq data) and the remainder, which we argue can be interpreted as the conditional entropy introduced at the post-transcriptional level. Our observations are qualitatively similar to those from a number of previous studies reporting widespread, regulated alternative TSS usage, often in a tissue-specific manner (Carninci *et al.*, 2006; Demircioğlu *et al.*, 2019; Forrest *et al.*, 2014), some of which have argued for a primary role of transcription relative to splicing (Pal *et al.*, 2011; Reyes and Huber, 2018). However, while the post-transcriptional entropy that we measure presumably derives primarily from splicing, it is worth noting that it could also be influenced by post-transcriptional up- or down-regulation of particular isoforms, e.g. through miRNA- or RBP-mediated decay. In some cases, post-transcriptional processes could even reduce entropy generated at the pre-RNA level, e.g. by sharply down-regulating particular pre-RNA isoforms relative to others. Importantly, this type of generation or reduction in entropy can only be detected if pre-RNA isoform diversity is independently characterized by a method like the one introduced here, rather than indirectly assessed from RNA-seq (or CAGE) data. For this reason, we believe our analysis is complementary to previous analyses of alternative promoters and TSSs.

There are a number of potential avenues for improvement of our current implementation of DENR. First, the method assumes a sum-of-squares loss function, which is equivalent to maximum-likelihood estimation under a Gaussian (or log normal, if optimized in log space) generating distribution for read-counts, with the counts for each bin assumed to be independent and identically distributed. Real nascent RNA-sequencing read-counts, however, tends to be not only overdispersed but non-uniform along the genome, with fairly pronounced spikes separated by intervals of reduced signal. The method could be extended to allow for maximum-likelihood estimation under an arbitrary generating distribution for read-counts, by making use of a general probabilistic model for nascent RNA-sequencing data that we have recently proposed (Siepel, 2021). This model could potentially accommodate autocorrelated read-counts along the genome sequence, although in this case, optimizing the mixture coefficients would become more complex and computationally expensive. Another advantage of this framework is that it would naturally accommodate a richer and more general model for changes in polymerase density along the gene-body, beyond the simple shape-profile correction introduced here. As a result, it might require a less heavy-handed masking strategy, by providing a better description for read-counts near TSSs and PASs. More work will be needed to determine if these generalizations are sufficiently advantageous to justify their complexity and computational costs.

A second limitation is that DENR effectively uses a ‘hard prior’ for candidate isoforms, either treating them as equally likely *a priori* or completely excluding them (i.e. assigning a prior probability of zero) based on the absence of a TSS prediction or other evidence of inactivity. A natural generalization would be to accept an arbitrary prior probability for each candidate isoform. These weights could potentially be determined based on a variety of relevant covariates, including not only TSS predictions but also, say, chromatin accessibility, chromatin contact, histone modification or RNA-seq data from a relevant cell type. The model would then combine the prior probabilities with the data likelihood to enable full Bayesian estimation of isoform abundances. A related extension would be to consider not only annotated isoforms but also ones suggested by the nascent RNA-sequencing data but not annotated. Such candidates could potentially be identified using a separate method [e.g. Anderson *et al.* (2020)] and given lower prior weights than annotated isoforms; if they had sufficient support in the data, they might still obtain high posterior probabilities.

Finally, the current inference method does not make use of a sparsity penalty to encourage the observed data to be explained using as few isoforms as possible. In initial experiments, we did not find that such penalties made a noticeable difference in our prediction performance, and in general, we do not observe a proliferation of isoforms with small weights. However, we do occasionally find that DENR gives high weights to short transcripts that happen to coincide with spikes in the data or pause peaks, apparently owing to a failure to account for spikes in the read-count data, as well as inadequacies in the shape-profile correction when applied to short isoforms. It is possible that a sparsity penalty—perhaps combined with the use of a richer model for read-counts—would help to eliminate some of these apparently spurious predictions.

Despite these limitations, we have shown that DENR is generally an effective tool for quantifying pre-RNA abundance at both the gene and isoform levels, with many possible downstream applications. We expect this method to be increasingly useful to the community as nascent RNA-sequencing data grow more abundant and are used for a wider variety of downstream applications.

## Supplementary Material

btab582_Supplementary_Data
