# Supplementary material for: Deconvolution of expression for nascent RNA-sequencing data (DENR) highlights pre-RNA isoform diversity in human cells
Source: Bioinformatics. 2021 Aug 11;37(24):4727–36. doi: 10.1093/bioinformatics/btab582 (PMC8665767; doi:10.1093/bioinformatics/btab582)
Supplement: btab582_Supplementary_Data [file btab582_supplementary_data.pdf]

Supplementary materials for

Deconvolution of Expression for Nascent RNA Sequencing Data (DENR) Highlights Pre-RNA Isoform Diversity in Human Cells

Yixin Zhao[1,*], Noah Dukler[1,*], Gilad Barshad[2], Shushan Toneyan[1], Charles G. Danko[2], and Adam Siepel[1]

[1]Simons Center for Quantitative Biology, Cold Spring Harbor Laboratory, Cold Spring Harbor, NY 11724, USA.

[2]Baker Institute for Animal Health, College of Veterinary Medicine, Cornell University, Ithaca, NY 14853, USA.

[*]These authors contributed equally to this work.

Supplementary methods

Human blood CD14$^+$ monocytes and CD4$^+$ T-cells isolation

Roughly 80 ml of human blood was drawn to and kept in spray-coated EDTA tubes (BD Vacutainer #366643) at 4°C. The following day, blood was diluted 1:1 in PBS and loaded on an equal volume of Ficoll-Paque (Fisher Scientific #45-001-750). Peripheral blood mononuclear cells (PBMCs) were then isolated by centrifugation for 20 min at 750xg. All layers, excluding the lower erythrocytes-containing layer, were moved to a new 50ml tube, and washed twice in PBS. PBMCs were then treated with 2 ml of erythrocytes lysis buffer (Lonza Walkersville inc. #120-02-070) for 1 min, flooded with 5 ml of RPMI supplemented with 10% FBS, and centrifuged for 10 min at 4°C and 1500 RPM on a Megafuge 40R Refrigerated Centrifuge (Thermo Scientific #75004518). CD14$^+$ monocytes were isolated from PBMCs by a magnetic cell separation system (MACS) using anti-human CD14 antibody attached to microbeads (Miltenyi Biotec #130050201) on an LS column (Miltenyi Biotec #130-042-401) following the manufacturer's protocol, while maintaining the flow-through of PBMCs without CD14$^+$ monocytes. CD4$^+$ T cells were then isolated from CD14$^+$ monocyte-free PBMCs using anti-human CD4 antibody attached to microbeads (Miltenyi Biotec #130045101) on a new LS column, following the same protocol. Finally, CD14$^+$ monocytes and CD4$^+$ T-cells were incubated for 1h of recovery in RPMI supplemented with 10% FBS before any downstream applications.

RNA-seq library preparation

Cells were flooded with 1 ml per 5×10^6 cells of TRI reagent (Molecular Research Center #TR118) and 0.2 ml of chloroform was added per 1 ml of TRI reagent followed by a vigorous vortexing for 20 sec. Cells were then incubated in the TRI reagent and chloroform solution for 2 minutes, followed by a 12,000xg centrifugation at 4°C for 15 min. The resulting aqueous phase was transferred to a new 1.5 ml tube and 0.5 ml of isopropyl alcohol was added for each 1 ml of TRI reagent initially used, and the solution was incubated in room temperature for 10 min, followed by a 10 min centrifugation in 12,000xg at 4°C. RNA was then washed twice with 75% ethyl alcohol, air-dried for 5 min with an open lid on ice and dissolved in DEPC-treated water. Poly-A enriched RNA-seq libraries were then prepared with up to 1 µg of isolated RNA, using the NEBNext® Ultra™ II Directional RNA Library Prep Kit for Illumina® (New England Biotech #E7760) with the NEBNext Poly(A) mRNA Magnetic Isolation Module (New England Biotech #E7490), following the manufacturer's protocol.

Nuclei isolation and PRO-seq library preparation

1×10^6 cells were centrifuged at 4°C for 5 min at 1000xg and washed twice with 1 ml of PBS. Cells were then re-suspended in 150 µl of wash buffer (10mM Tris-Cl pH 8.0, 300mM sucrose, 10mM NaCl, 2mM $MgAc_2$, 2.5 µM DTT, 1X protease inhibitor cocktail (Thermo Scientific, #A32965)) supplemented with 0.6U of SUPERase In RNase Inhibitor (Thermo Fisher Scientific, #AM2696). 150 µl of 2X lysis buffer (10mM Tris-Cl pH 8.0, 300mM sucrose, 10mM NaCl, 2mM $MgAc_2$, 6mM $CaCl_2$, 0.2% NP-40) were added to the solution and samples were gently pipetted up and down 10 times, to facilitate nuclei release. Released nuclei were then centrifuged at 4°C for 5 min at 1000xg, washed with 1 ml of a 1:1 ratio solution of wash buffer and 2X lysis buffer and re-suspended in 50 µl of storage buffer (50mM Tris-CL pH 8.3, 40% glycerol, 5mM $MgCl_2$, 0.1 mM EDTA, 2.5 µM DTT, 1X protease inhibitor cocktail) supplemented with 0.2U of SUPERase In RNase Inhibitor. PRO-seq run-on and library preparation was completed following a recently updated protocol (Judd *et al.*, 2020). Briefly, nuclei were run-on by incubating at 37°C for 5 min in run-on buffer (10 mM Tris-Cl pH 8.0, 5 mM $MgCl_2$, 1 mM DTT, 300 mM KCl, 40 µM Biotin-11-CTP, 40 µM Biotin-11-UTP, 40 µM Biotin-11-ATP, 40 µM Biotin-11-GTP, 1% (w/v) Sarkosyl in DEPC H_2O). The run-on reaction was stopped by adding Trizol LS (Life Technologies, #10296-010). RNA was

pelleted with the addition of GlycoBlue (Ambion, #AM9515) to visualize the pellet, re-suspended in diethylpyrocarbonate (DEPC)-treated water and heat denatured for 40 sec. RNA was digested using 0.2N NaOH on ice for 6 min, which yields RNA lengths ranging from ∼20–500 bases. The 3′ adapter (sequence: AGATCGGAAGAGCACACGTCTGAACTC) was ligated using T4 RNA Ligase 1 (NEB, M0204L) and purified nascent RNA using streptavidin beads (NEB, S1421S). Next the nascent RNA was decapped using RppH (NEB, M0356S), the 5′ end was phosphorylated using T4 polynucleotide kinase (NEB, M0201L), and the 5′ adapter (sequence: GTTCAGAGTTCTACAGTCCGACGATC) was ligated. RNA was removed from the beads and a reverse transcription was performed using Superscript IV Reverse Transcriptase (Life Technologies) and amplified using Q5 High-Fidelity DNA Polymerase (NEB, M0491L). PRO-seq libraries were sequenced using the NextSeq500 high-throughput sequencing system (Illumina) at the Cornell University Biotechnology Resource Center.

Abundance estimation for newly generated PRO-seq and RNA-seq data

For mature RNA abundances in RNA-seq, adapters (sequence: AGATCGGAAGAGC) in raw reads were first removed using Cutadapt (v2.10) (Martin, 2011). Clean reads were then aligned to the human genome using HISAT2 (v2.2.1) (Kim *et al.*, 2019). Mature RNA isoform abundances were quantified using StringTie (v2.1.4) (Pertea *et al.*, 2016). Human genome (GRCh38.p13) and GTF files were downloaded from Ensembl (release 99) (Cunningham *et al.*, 2019). For pre-RNA isoform abundances, PRO-seq libraries were first processed by the PROseq2.0 pipeline using paired-end mode (Chu *et al.*, 2019), then DENR was used to quantify abundances. The shape-profile correction, log-transformation of read-counts, and masking of one bin around the TSS and four bins around the PAS were used, as in other analyses, but in this case, inactive isoforms were identified as those not detected in the RNA-seq data rather than by using DENR's TSS predictions. 10,650 genes with abundance estimates > 0 in $CD4^+$ T cell and $CD14^+$ monocyte samples were used for the entropy calculation.

References

Chu, T. *et al.* (2019). Discovering Transcriptional Regulatory Elements From Run-On and Sequencing Data Using the Web-Based dREG Gateway. *Curr Protoc Bioinformatics*, **66**(1), e70.

Cunningham, F. *et al.* (2019). Ensembl 2019. *Nucleic Acids Res*, **47**(D1), D745–D751.

Judd, J. *et al.* (2020). A rapid, sensitive, scalable method for precision run-on sequencing (pro-seq). *bioRxiv*.

Kim, D. *et al.* (2019). Graph-based genome alignment and genotyping with HISAT2 and HISAT-genotype. *Nat Biotechnol*, **37**(8), 907–915.

Martin, M. (2011). Cutadapt removes adapter sequences from high-throughput sequencing reads. *EMBnet.journal*, **17**(1), 10–12.

Pertea, M. *et al.* (2016). Transcript-level expression analysis of RNA-seq experiments with HISAT, StringTie and Ballgown. *Nat Protoc*, **11**(9), 1650–1667.

Supplementary tables

Transcript ID	Model name	Abundance
ENST00000393446<br>ENST00000393451<br>ENST00000323984<br>ENST00000393449<br>ENST00000446490	G14406M1	29.6
ENST00000432298<br>ENST00000422922	G14406M6	42.9

Table S1: **DENR isoform-abundance estimates for *ST7***

Gene name	Transcript ID	Model name	Abundance
SEC22C	ENST00000423701<br>ENST00000273156<br>ENST00000449617<br>ENST00000264454	G10933M2	60.5
	ENST00000450981	G10933M6	8.3
SS18L2	ENST00000447630	G10431M1	30.6
	ENST00000011691<br>ENST00000474941	G10431M2	70.3
NKTR	ENST00000232978<br>ENST00000429888<br>ENST00000617821	G10432M1	132.1
	ENST00000460910	G10432M3	4.6
	ENST00000459950	G10432M5	29.1

Table S2: **DENR isoform-abundance estimates for *SEC22C*, *SS18L2* and *NKTR***

Supplementary figures

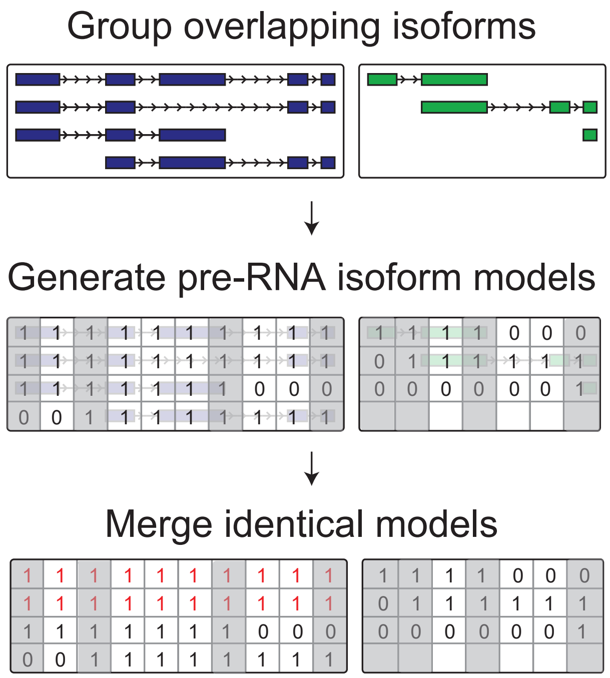

Figure S1: **Processing isoform annotations for DENR.** This workflow shows the steps involved in generating pre-RNA isoform models from mature RNA isoform annotations. Mature RNA isoform annotations were first downloaded from a database, then grouped into clusters. Bins were overlaid on each cluster (default bin size: 250bp), and presence or absence of annotations in each of these bins was recorded in a design matrix. A user-selected number of bins were optionally masked at the start and end of each isoform. Finally, identical pre-RNA isoforms after masking were merged. Here, the first two mature-RNA isoforms of the blue gene are identical at the pre-RNA level (highlighted in red) and would be merged.

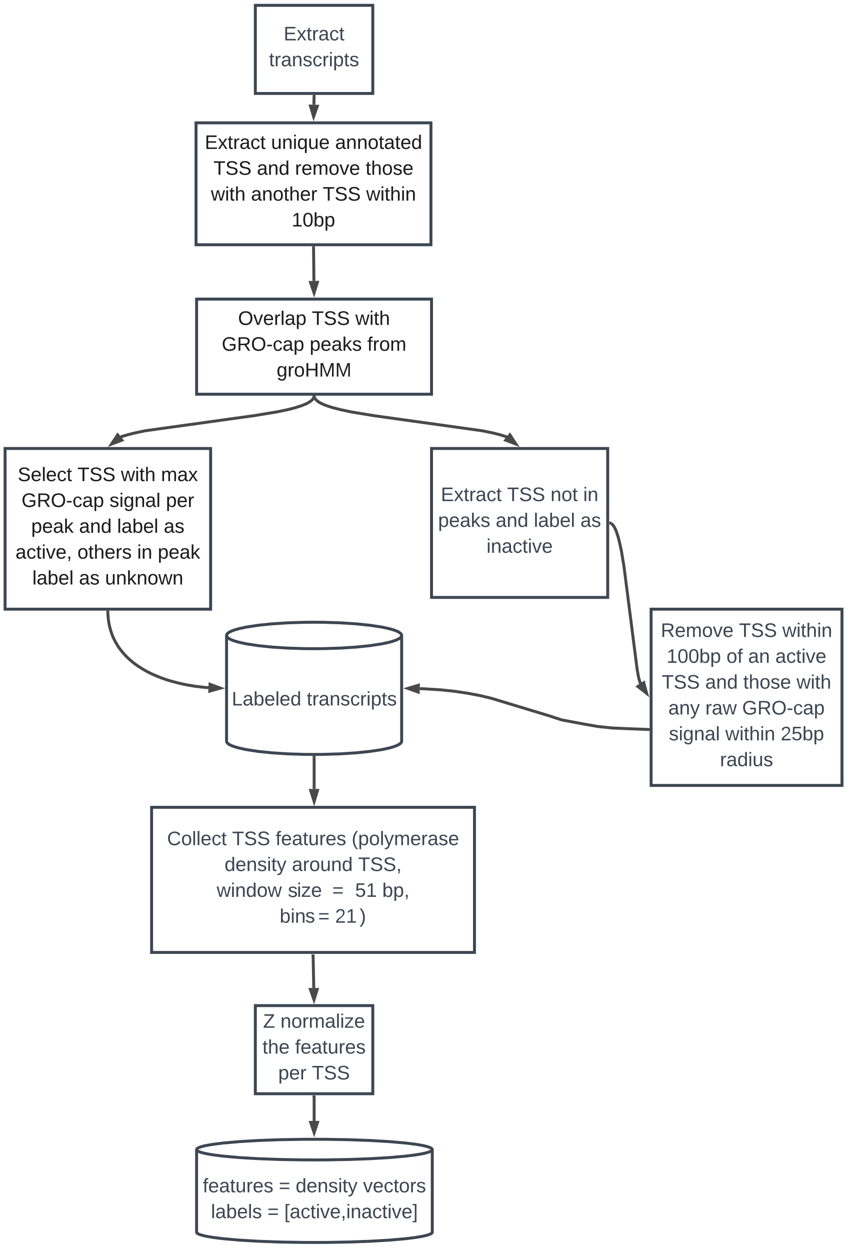

Figure S2: **Workflow for annotating training data for the TSS classifier.** TSS annotations were retrieved from Ensembl. To ensure the polymerase signal was uniquely attributable to a single TSS, all TSSs with another TSS within 10bp were removed. This set of TSSs was then overlapped with groHMM peaks called from cell type matched GRO-cap data. TSSs were labeled as "active" if they overlapped with GRO-cap peaks. If a single GRO-cap peak overlapped with multiple TSSs, the TSS with max GRO-cap signal was selected. The other TSSs located in the same peak were labeled as unknown; in addition, TSSs not overlapped by any GRO-cap peaks were labelled as "inactive." If these TSSs were near an active TSS (distance < 100bp) and there was any raw GRO-cap signal (distance < 25bp) nearby, they were treated as being "unknown". Polymerase density was then collected as TSS features, with 21 bins around TSS (window size $= 51$bp). Examples with only zero-valued entries were removed from the data set. The feature vectors were row-wise Z-normalized and fed into the CNN for model training with the corresponding labels.

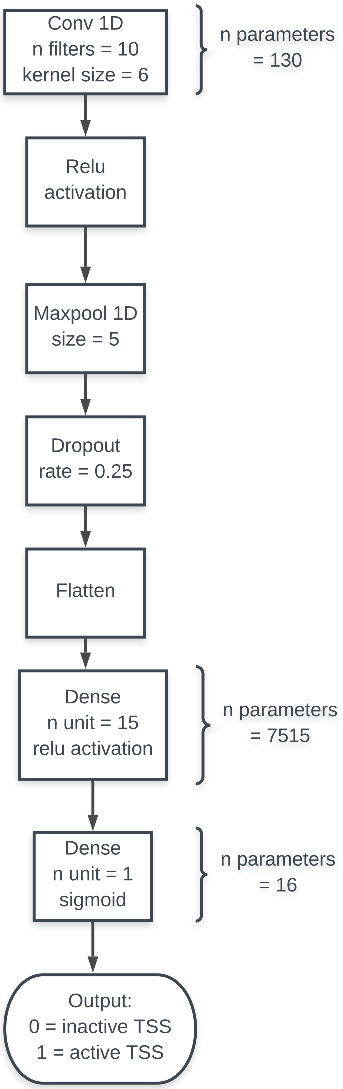

Figure S3: **Architecture of convolutional neural network for identifying active TSSs.** The CNN for classifying TSS activity is composed of a single 1-D convolutional layer followed by a ReLu activation function and max-pooling. The output from the max pooling layer was then flattened and fed into a densely connected layer and finally a single sigmoid output to classify the TSS.

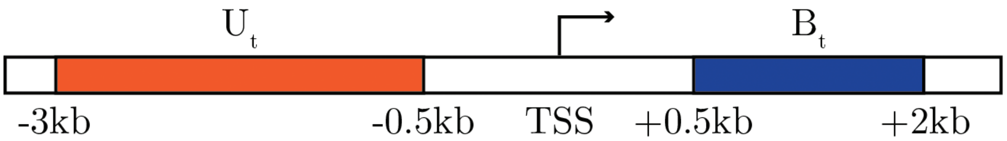

$$UPR = \frac{B_t \;/\; Length_B + 10^{-3}}{U_t \;/\; Length_U + 10^{-3}}$$

Figure S4: **A heuristic method for identifying active isoforms.** Because the TSS predictor misses some active TSSs with abnormal polymerase density patterns, an additional heuristic is used to rescue them. We calculate an upstream polymerase ratio (UPR) using the regions indicated in the schematic above. B_t and U_t are the read counts within the blue and orange regions, while $Length_B$ and $Length_U$ are their lengths (1.5 kb and 2.5 kb, respectively). If the UPR of an isoform is ≥ 5 and there are no other active isoforms within 5 kb upstream or 6 kb downstream of its TSS, then this isoform is eligible to be assigned a non-zero weight during the fitting step of the algorithm.

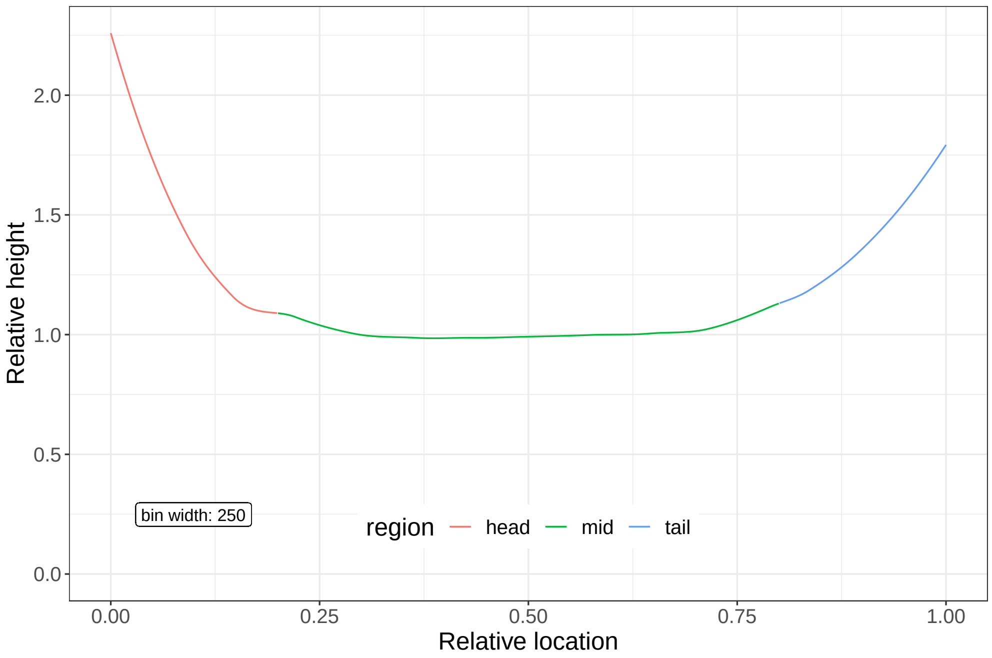

Figure S5: **Shape profile of PRO-seq signal across isoform annotations** A designated set of isoforms was used for the shape-profile correction in K562 cells (n=986). The first and last 3 kbp of each isoform were mapped proportionally to the intervals [0, 0.2] and [0.8, 1], respectively, and the remaining portion was mapped to the (0.2, 0.8) interval.

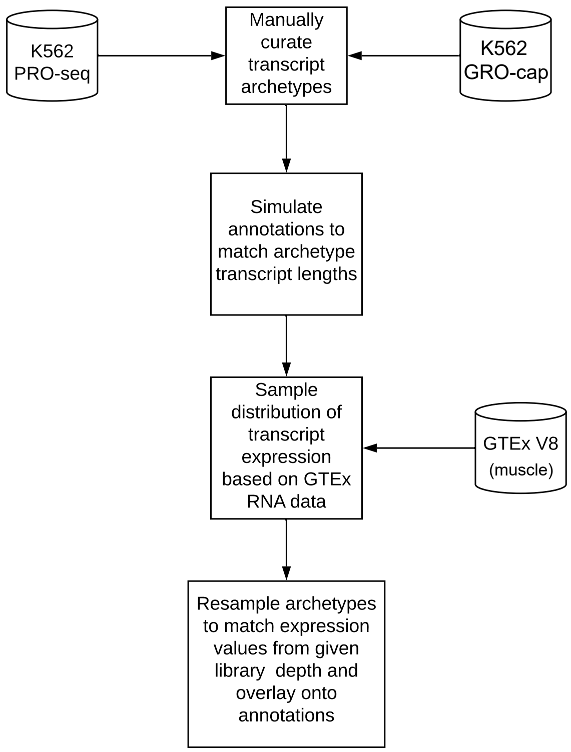

Figure S6: **Algorithm for empirically simulating nascent RNA sequencing data.** The algorithm is described in the **Methods** section.

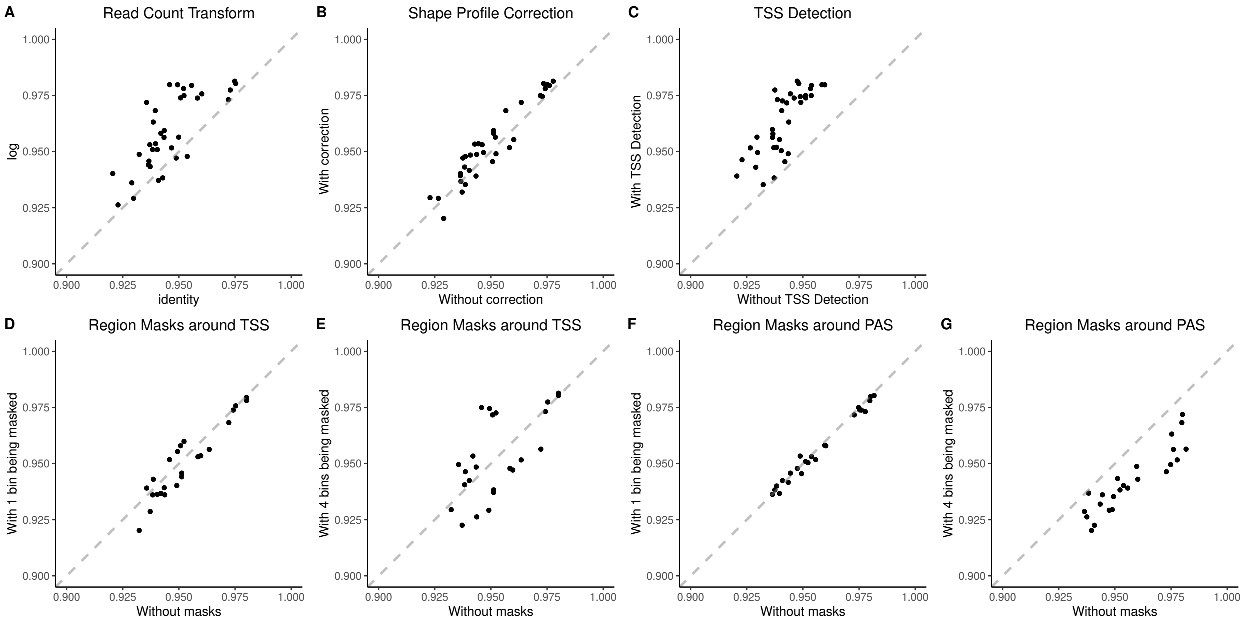

Figure S7: **Benchmarking of DENR with various combinations of optional features (gene-level).** DENR was run with various numbers (0, 1, or 4) of bins masked at the TSS and PAS of each isoform, and with or without log-transformation of read-counts, shape-profile correction, and TSS prediction ($3 \cdot 3 \cdot 2 \cdot 2 \cdot 2 = 72$ combinations in total). Pearson's correlation coefficients (r) were calculated between DENR estimates and true values at the gene level. Paired comparisons are made within each optional feature and Wilcoxon tests were performed. (**A**) Read count transform (p=4.07e-4) (**B**) Shape profile correction (p=0.269) (**C**) TSS detection (p=1.93e-9) (**D-G**) 1 or 4 bins masked around TSS and PAS compared with no masking (p values are 0.690, 0.943, 0.846 and 0.00138 for (**D**) to (**G**), respectively).

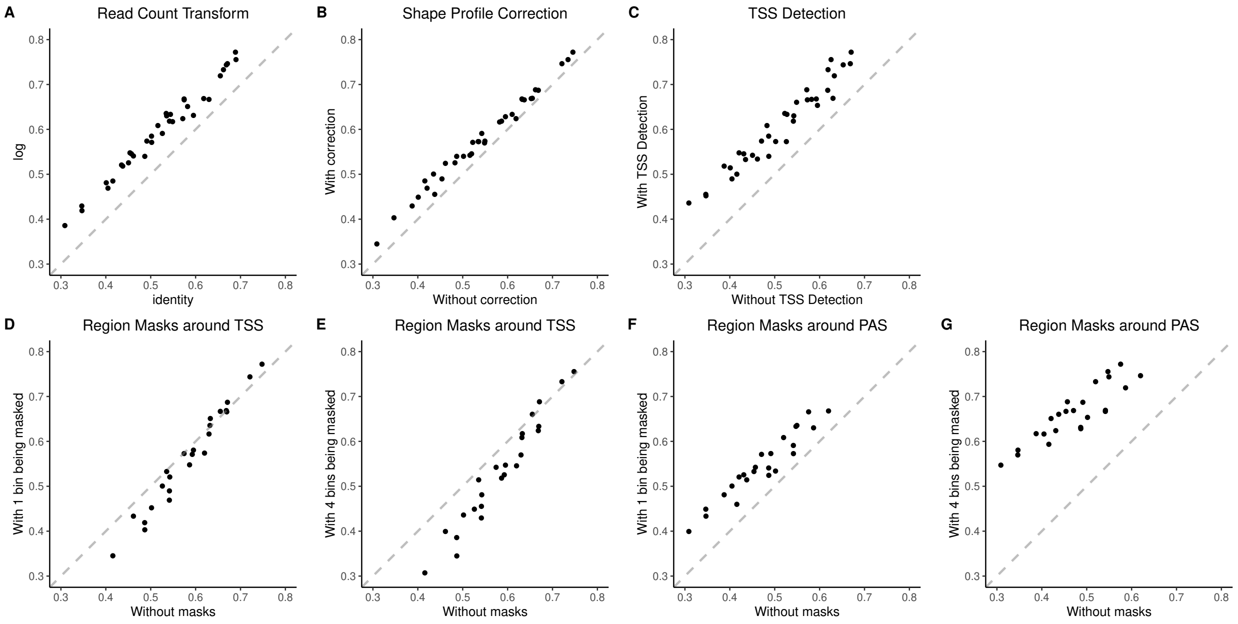

Figure S8: **Benchmarking of DENR with various combinations of optional features (isoform-level).** DENR was run with various numbers (0, 1, or 4) of bins masked at the TSS and PAS of each isoform, and with or without log-transformation of read-counts, shape-profile correction, and TSS prediction ($3 \cdot 3 \cdot 2 \cdot 2 \cdot 2 =$ 72 combinations in total). Pearson's correlation coefficients (r) were calculated between DENR estimates and true values at the isoform level. Paired comparisons are made within each optional feature and Wilcoxon tests were performed. (**A**) Read count transform (p=0.0036) (**B**) Shape profile correction (p=0.149) (**C**) TSS detection (p=1.35e-4) (**D-G**) 1 or 4 bins masked around TSS and PAS compared with no masking (p values are 0.533, 0.115, 0.00339 and 1.69e-11 for (**D**) to (**G**), respectively).

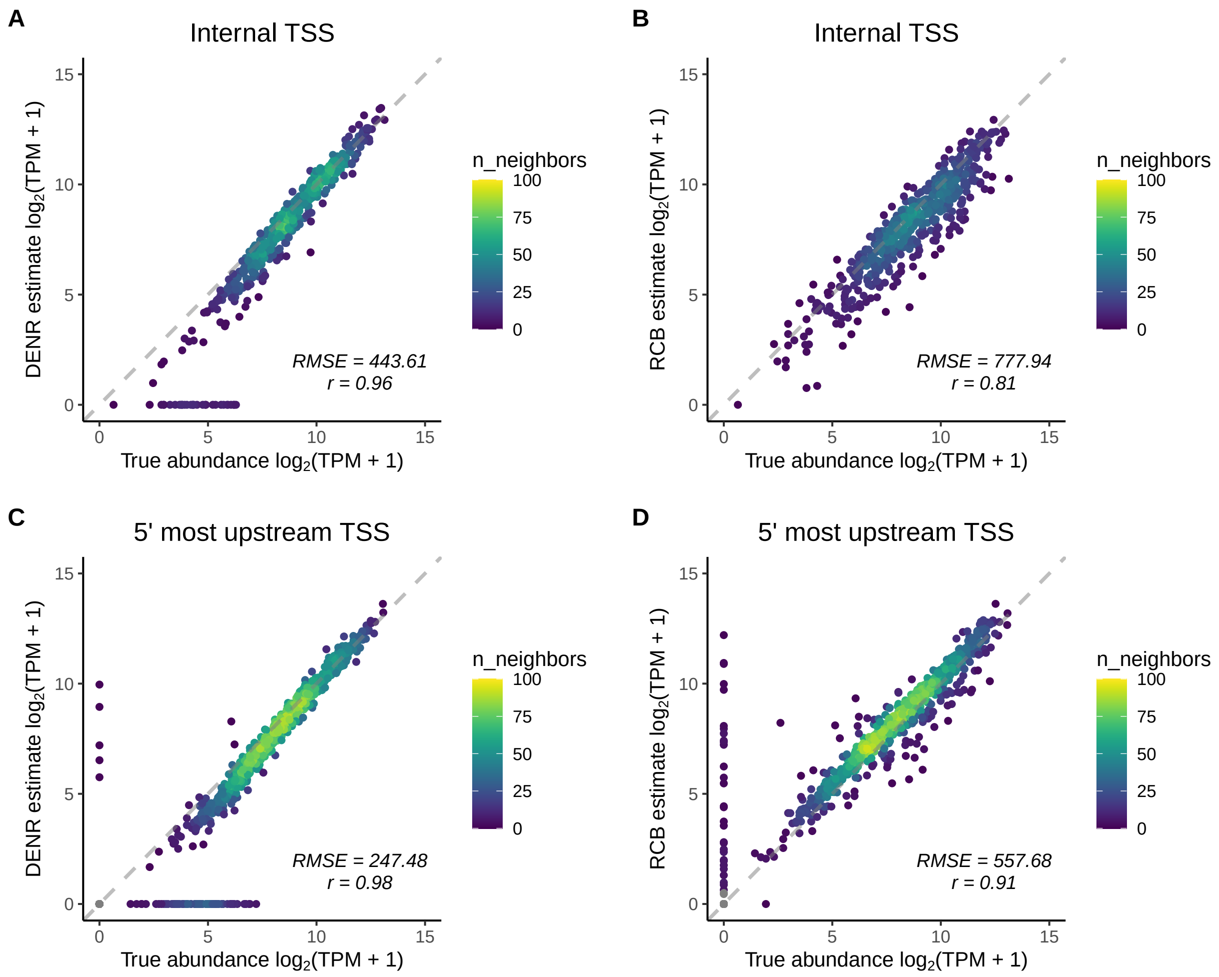

Figure S9: **Comparison of DENR and the RCB method on subsets of genes of interest.** **(A)** DENR estimates for genes whose dominant isoform corresponds to an internal TSS. **(B)** RCB estimates for genes whose dominant isoform corresponds to an internal TSS. **(C)** DENR estimates for genes whose dominant isoform corresponds to the $5'$-most upstream TSS. **(D)** RCB estimates for genes whose dominant isoform corresponds to the $5'$-most upstream TSS. RMSE = root-mean-square error, r = Pearson's correlation coefficient.

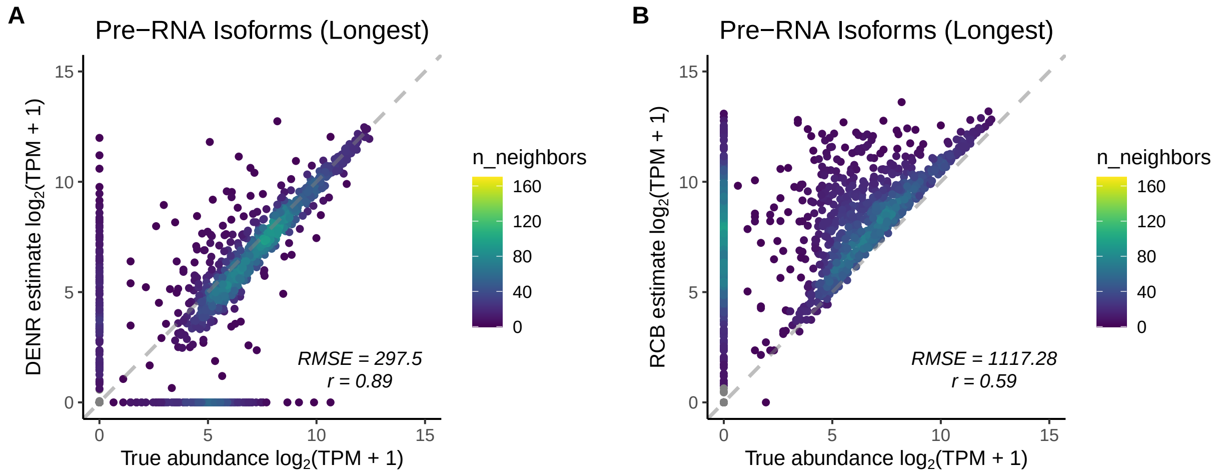

Figure S10: **Comparison of DENR and the RCB method for quantifying nascent RNA abundance for longest isoforms.** True (x-axis) vs. estimated (y-axis) abundance at the longest isoform levels, based on 1500 simulated loci. Data were simulated using nascentRNASim, which resamples real PRO-seq read counts and assumes a distribution of relative isoform abundances derived from real RNA-seq data. RMSE = root-mean-square error, r = Pearson's correlation coefficient.

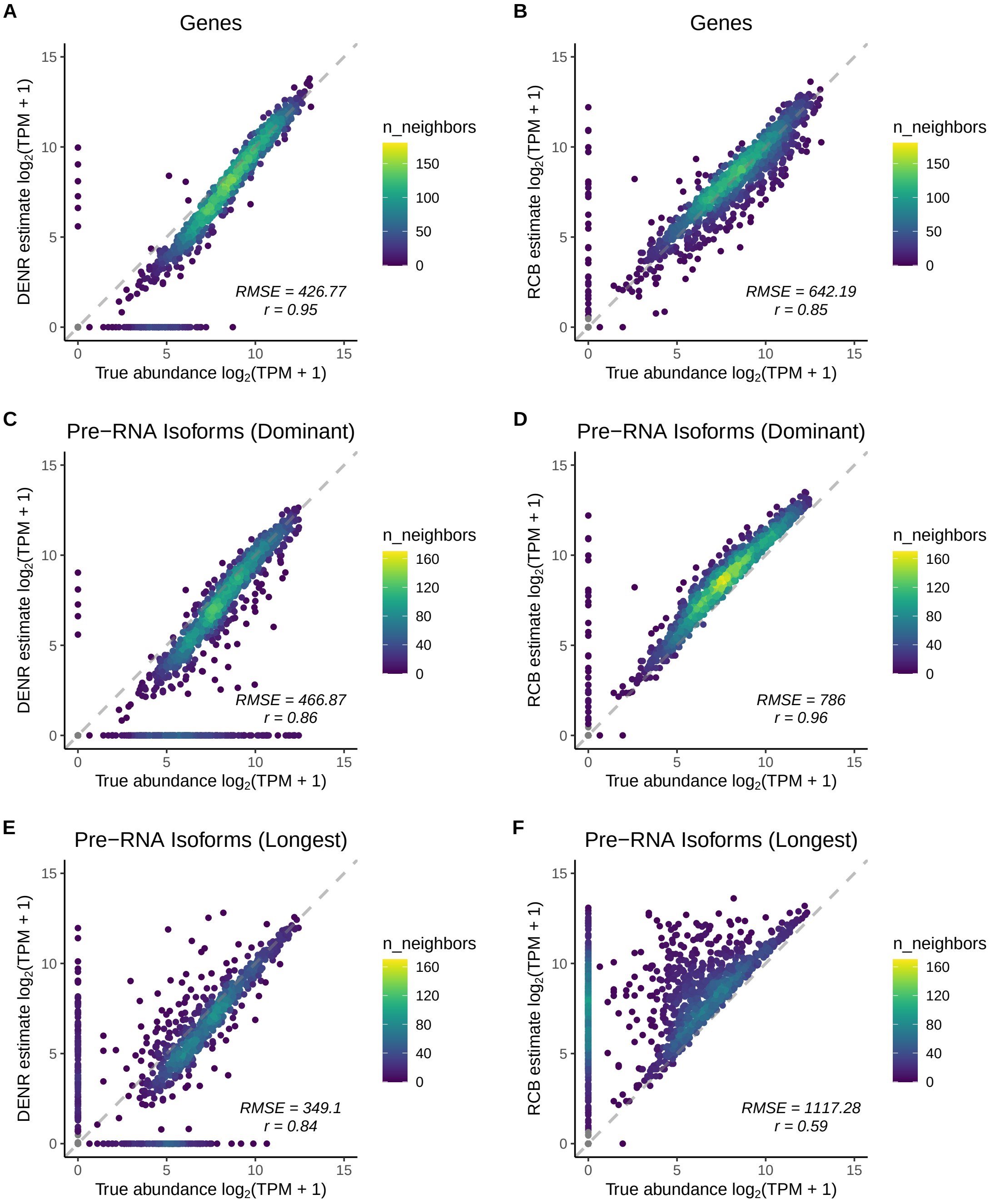

Figure S11: **Comparison of DENR and the RCB method for quantifying nascent RNA abundance with an alternative bin size of 125 bp.** All other parameters are the same as those in **Fig. 2**. True (x-axis) vs. estimated (y-axis) abundance at the gene (**A & B**) and the isoform (**C–F**) levels, based on 1500 simulated loci. Data were simulated using nascentRNASim, which resamples real PRO-seq read counts and assumes a distribution of relative isoform abundances derived from real RNA-seq data. Results are shown for both the "dominant" (most highly expressed) isoform (panels **C&D**) and the longest isoform (panels **E&F**). RMSE = root-mean-square error, r = Pearson's correlation coefficient.

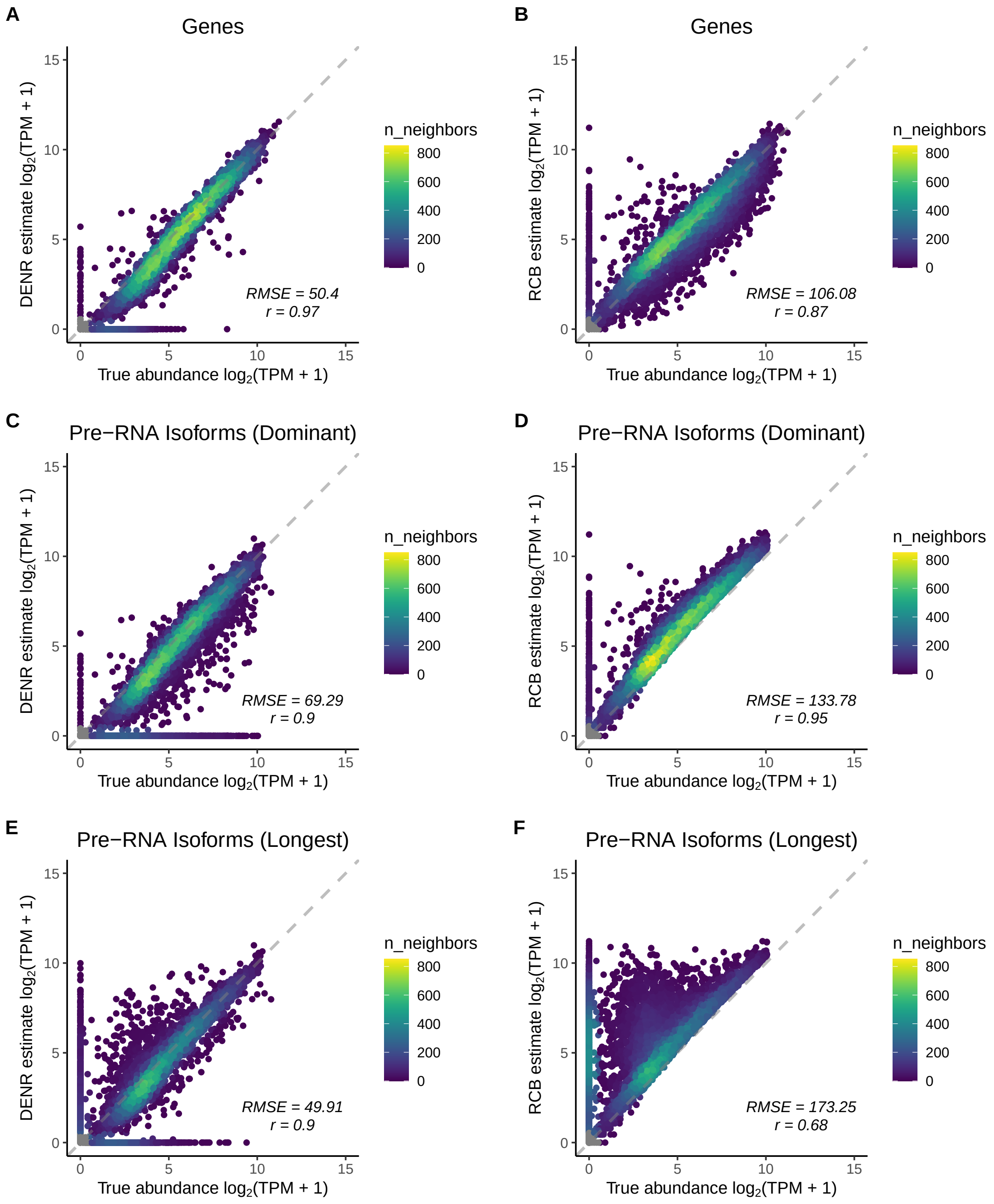

Figure S12: **Comparison of DENR and the RCB method for quantifying nascent RNA abundance based on a larger simulated data set.** All parameters used in DENR here are the same as those in **Fig. 2** except simulated data set contains 10,000 loci generated from 145 archetypes. True (x-axis) vs. estimated (y-axis) abundance at the gene (**A & B**) and the isoform (**C–F**) levels. Results are shown for both the "dominant" (most highly expressed) isoform (panels **C&D**) and the longest isoform (panels **E&F**). RMSE = root-mean-square error, r = Pearson's correlation coefficient.

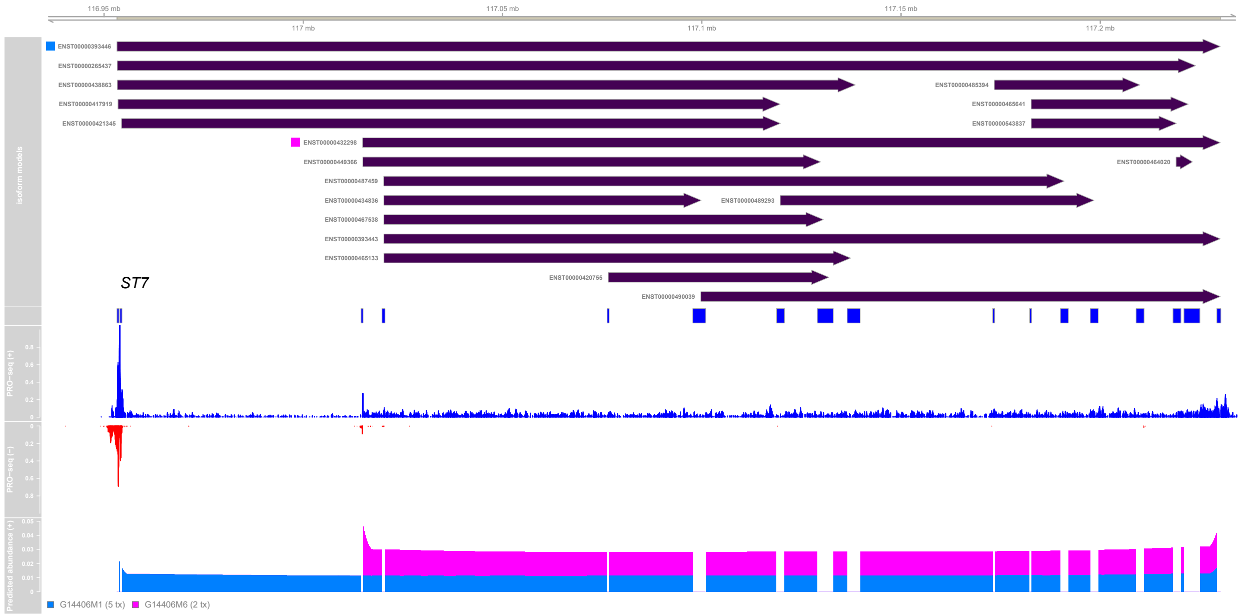

Figure S13: **DENR abundance estimation for pre-RNA isoforms of *ST7* in K562 cells.** The *ST7* (suppression of tumorigenicity 7; ENSG00000004866) gene has 30 isoform annotations in Ensembl, which DENR merges into 19 distinct pre-RNA isoform models (bars at *top*). Based on the observed PRO-seq data (*middle*, in blue and red), DENR estimates nonzero abundances for only two of these isoforms (marked in light blue and pink). The plot at *bottom* shows the expected relative contribution of each isoform model to the overall read counts per bin. Notice the effect of the shape-profile adjustment near the 5′ and 3′ ends. Notice also that the PRO-seq data reveals bidirectional transcription near the TSSs of both active isoforms; these signals are used by the machine-learning predictor to help identify sequence reads associated with these isoforms.

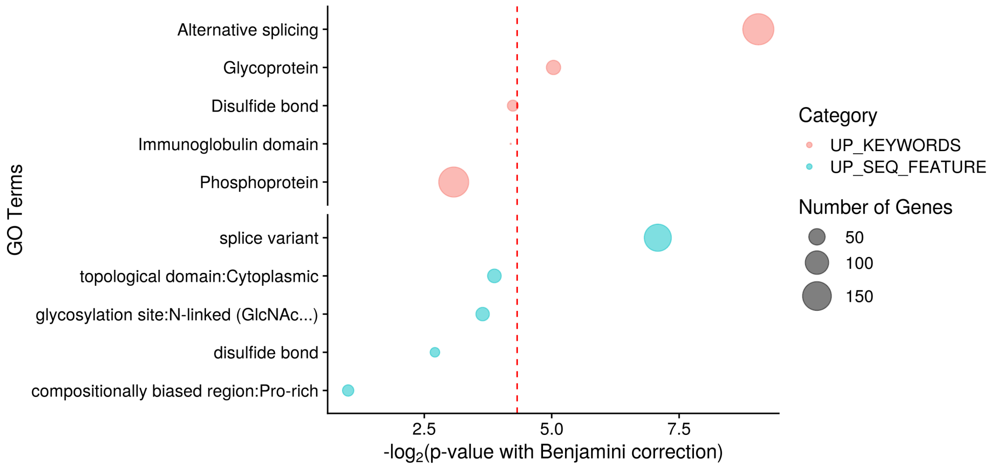

Figure S14: **Gene Ontology analysis for genes that make use of different TSSs in K562 and CD4$^+$ T cells**. Gene Ontology enrichment for UniProt keywords and sequence annotations with the five smallest *p*-values are shown.

A

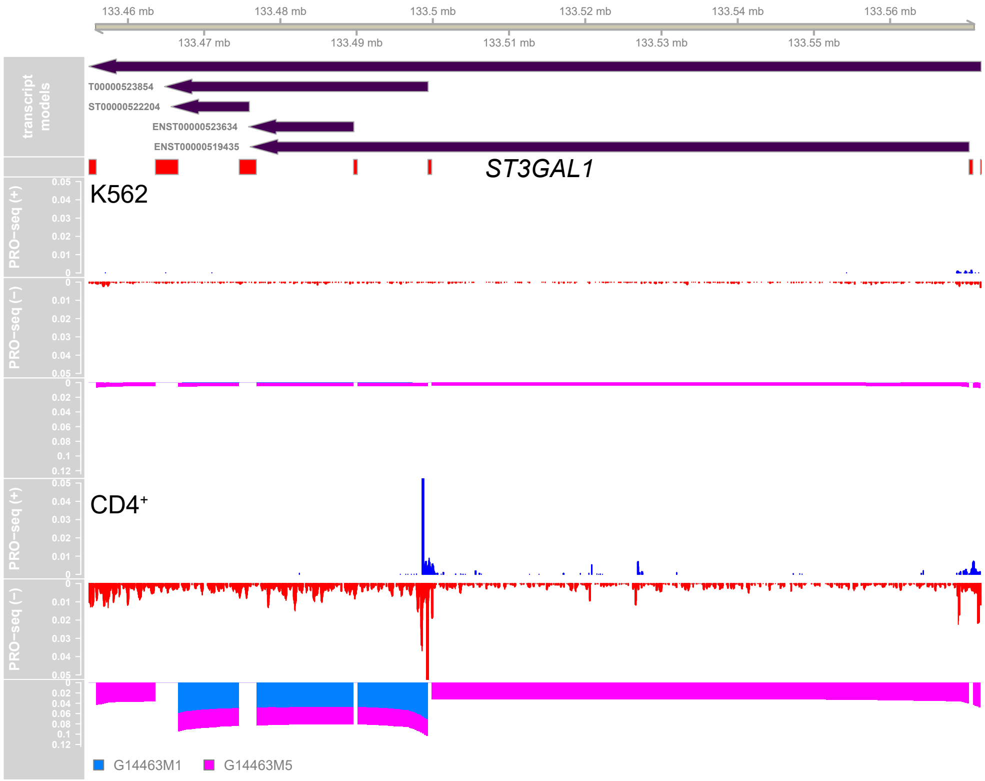

B

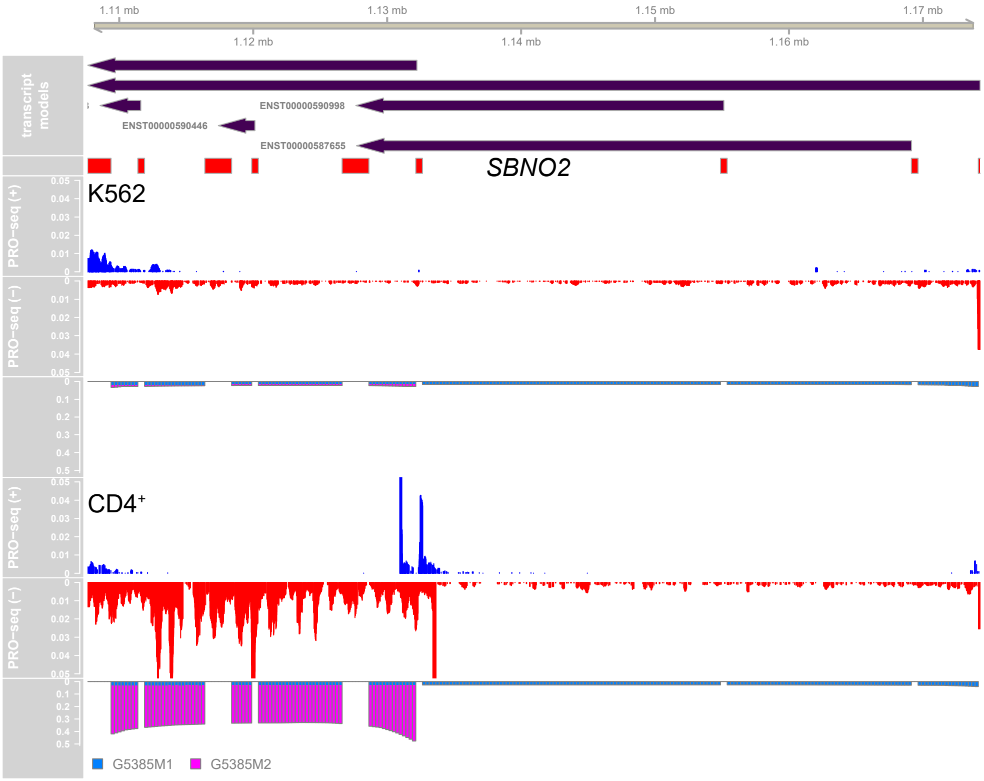

Figure S15: **Cell type specific TSS usage.** Additional examples for genes using different TSSs in K562 and $CD4^+$ T cells. **(A)** *ST3GAL1* **(B)** *SBNO2* **(C)** *PPP2R5C* **(D)** *CLCN3* **(E)** *FBXL5* **(F)** *CCM2*

C

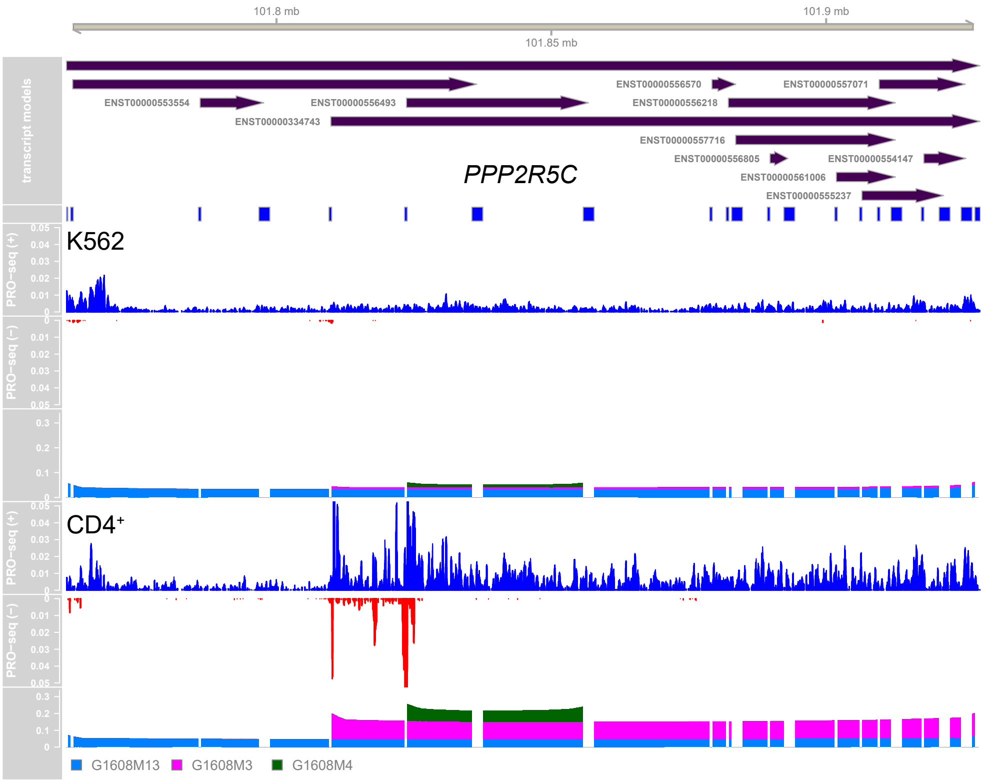

D

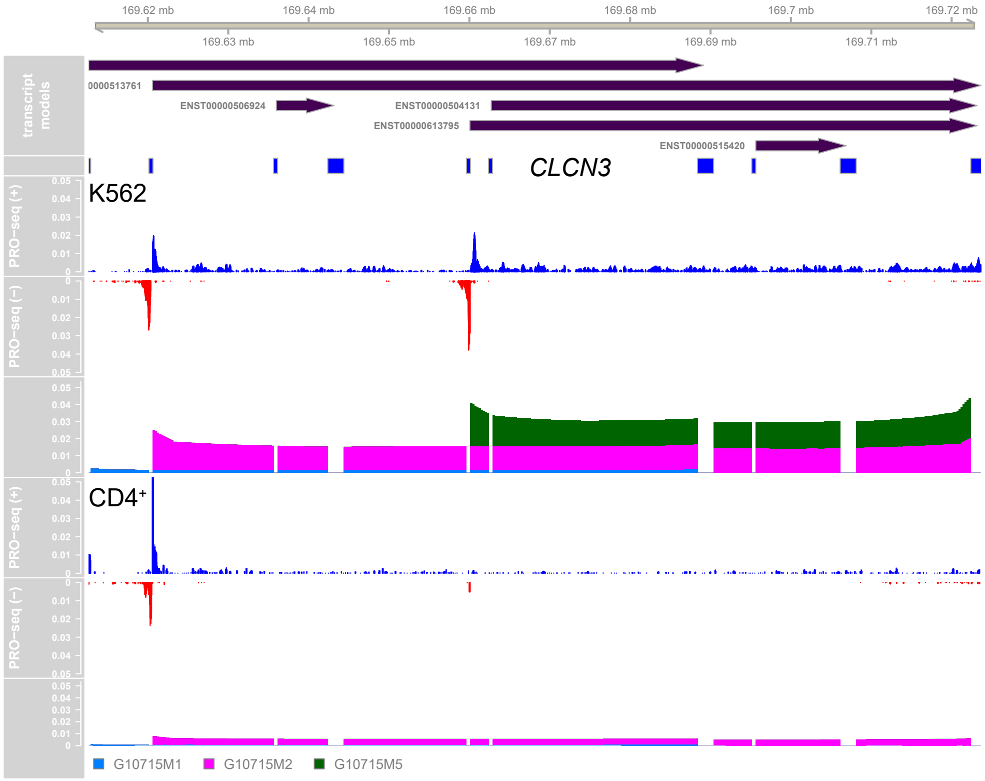

Figure S15: **Cell type specific TSS usage.** Additional examples for genes using different TSSs in K562 and $CD4^+$ T cells. **(A)** *ST3GAL1* **(B)** *SBNO2* **(C)** *PPP2R5C* **(D)** *CLCN3* **(E)** *FBXL5* **(F)** *CCM2*

E

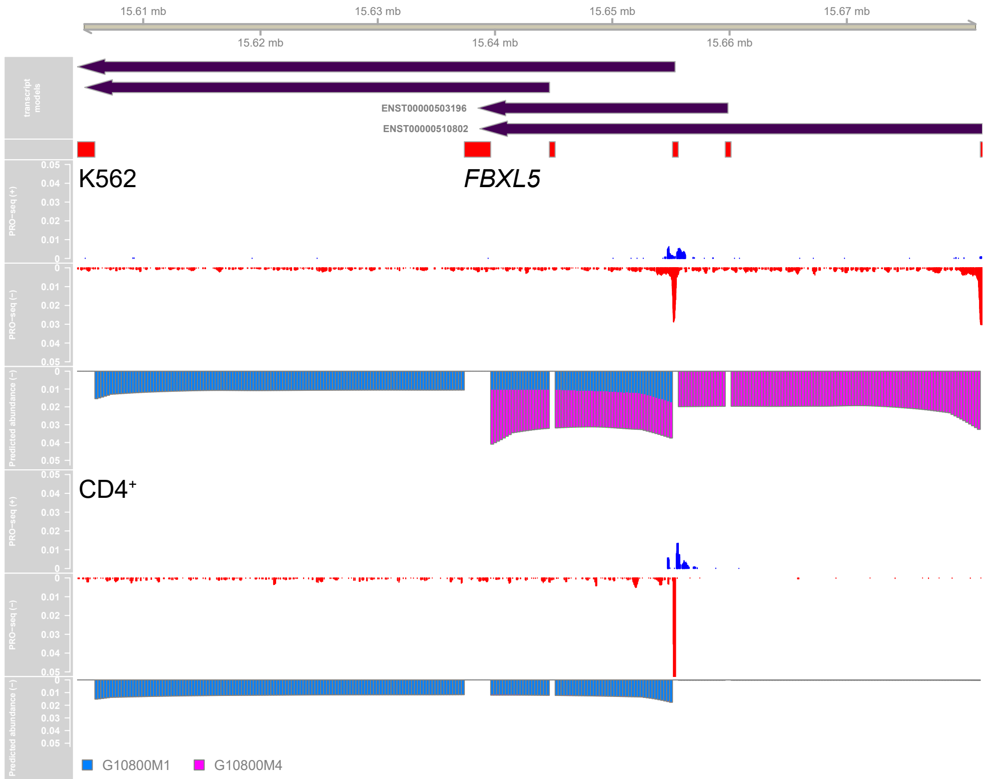

F

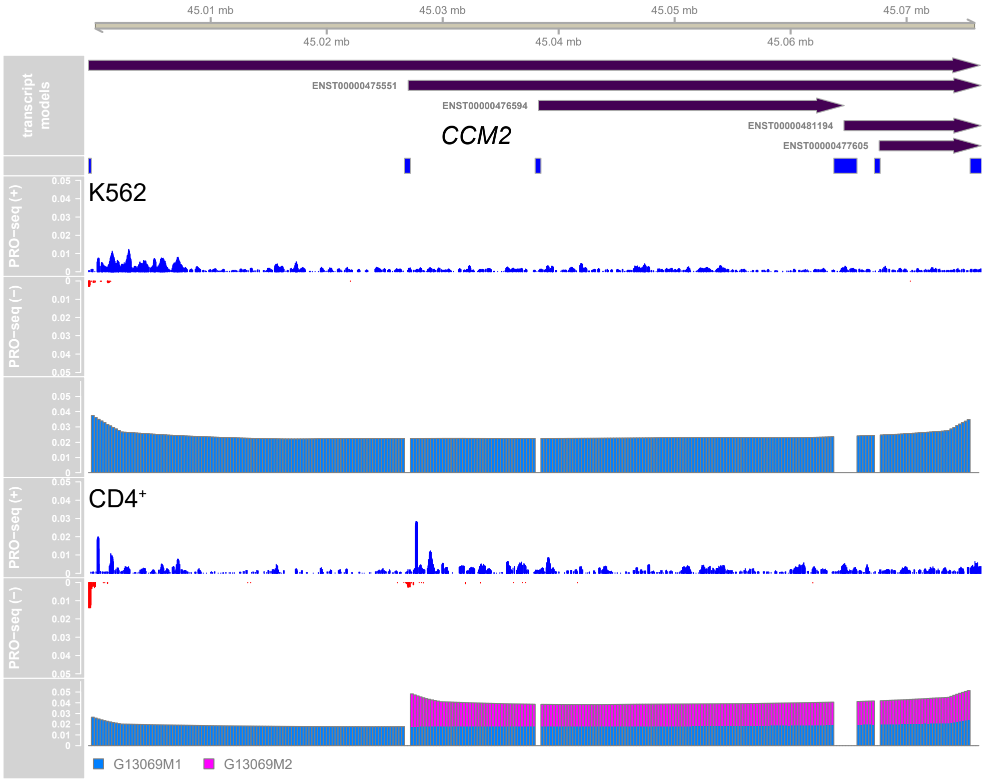

Figure S15: **Cell type specific TSS usage.** Additional examples for genes using different TSSs in K562 and $CD4^{+}$ T cells. **(A)** *ST3GAL1* **(B)** *SBNO2* **(C)** *PPP2R5C* **(D)** *CLCN3* **(E)** *FBXL5* **(F)** *CCM2*

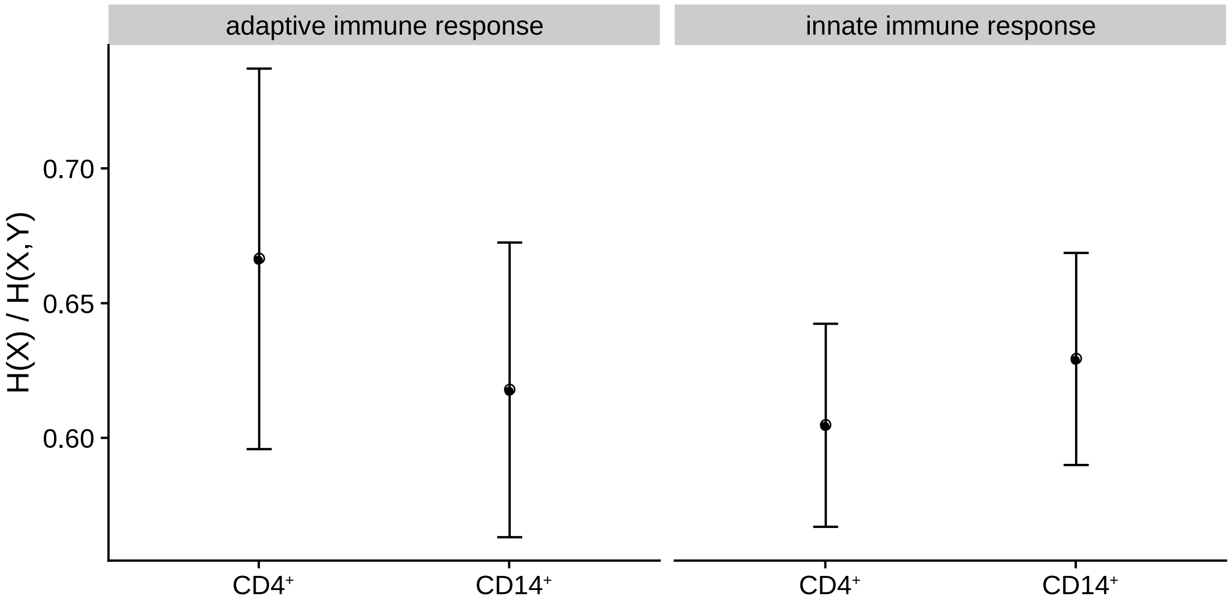

Figure S16: **Fractional contribution from primary transcription to isoform diversity for immune genes.** Genes are associated with the Gene Ontology terms "adaptive immune response" (GO:0002250; $n = 116$) and "innate immune response" (GO:0045087; $n = 287$). Error bars represent the standard deviation of the mean as estimated by bootstrap resampling ($n = 100$). The fraction was somewhat elevated in adaptive-immunity-related genes in $CD4^+$ T cells (Wilcoxon signed-rank test, p=0.0002), and slightly elevated in innate-immunity-related genes in $CD14^+$ monocytes (Wilcoxon signed-rank test, p=8.59e-14).

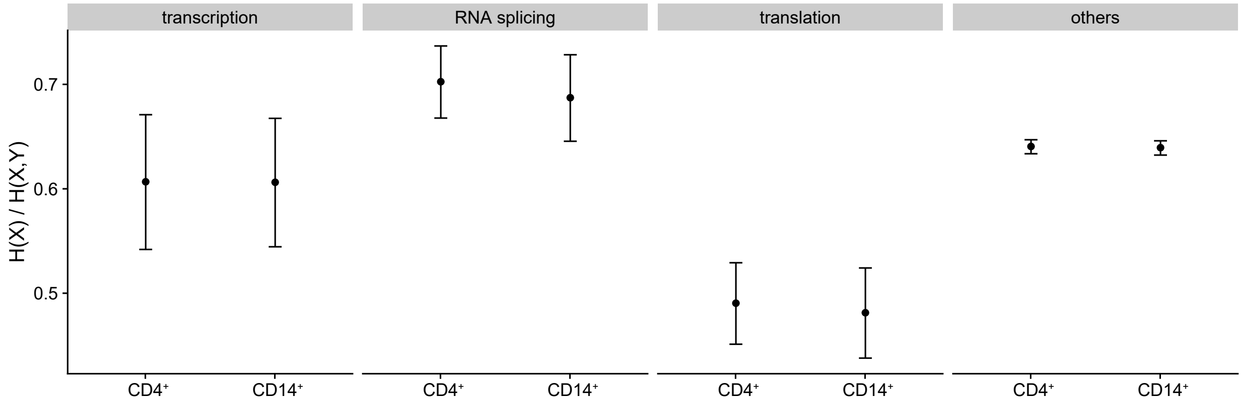

Figure S17: **Fractional contribution from primary transcription to isoform diversity for genes in different categories.** Genes are associated with the Gene Ontology terms "transcription, DNA-templated" (GO:0006351; $n = 88$), "RNA splicing" (GO:0008380; $n = 245$), "translation" (GO:0006412; $n = 226$) and all other genes not in these terms ($n = 10095$). Error bars represent the standard deviation of the mean as estimated by bootstrap resampling ($n = 100$).
